# Supplementary material for: Protect or prevent? A practicable framework for the dilemmas of COVID-19 vaccine prioritization
Source: PLoS One. 2025 Jan 22;20(1):e0316294. doi: 10.1371/journal.pone.0316294 (PMC11753641; doi:10.1371/journal.pone.0316294)
Supplement: S2 Appendix — (PDF) [file pone.0316294.s002.pdf]

Protect or prevent? A practicable framework for the dilemmas of COVID-19 vaccine prioritization
Supporting Information

Raghu Arghal[1*], Harvey Rubin[2], Shirin Saeedi Bidokhti[1], Saswati Sarkar[1]

March 2023

1 Department of Electrical and Systems Engineering, University of Pennsylvania, Philadelphia, PA, United States

2 Division of Infectious Diseases, Department of Medicine, University of Pennsylvania School of Medicine, Philadelphia, PA, United States

***** Corresponding Author (rarghal@seas.upenn@edu)

2 Generalizations of the basic model

For simplicity of exposition, in the main body we presented a bare bones model in which 1) vaccinated or recovered individuals are not infected, and 2) vaccine has only one dose. In truth, vaccinated and recovered individuals can be infected, albeit with lower rates of infection, symptomatic infection, hospitalization, and death [9, 10]. Also, while some vaccines have one dose, others need two doses [11]. We generalize our model to consider 1) infections after vaccination and recovery (Section 2.1) 2) multi dose vaccines (Section 2.2) and 3) several public health objectives of interest other than overall death count (Section 2.3). The ease of these generalizations demonstrate the flexibility of our model. These generalizations were used throughout our numerical investigation to compute and evaluate the optimal and near-optimal vaccination strategies in Section 4. Similarly, one may allow for vaccination of exposed or infected individuals, ongoing booster vaccines, or other circumstances.

2.1 Breakthrough infections and reinfection

We now consider that vaccinated individuals can be infected, though with lower rates of infection, symptomatic infection, hospitalization, and death [9, 10]. Thus, the disease spread rate is lower when at least one of the individuals in the pair involved in transmitting the virus is vaccinated than when neither is vaccinated, transition rates to asymptomatic states are higher for the vaccinated and transition rates to pre-symptomatic, hospitalized, dead are lower for the vaccinated. To allow for the differences in these rates for the vaccinated individuals, a new set of exposed, pre-symptomatic, asymptomatic, early stage infection, late stage infection, hospitalized are created for the vaccinated (Figure S1). The ODE representation of the state dynamics is similar to Figure 1, with the difference that there are now additional states, additional differential equations and additional quadratic terms (i.e. additional red terms) in the differential equations representing disease transmission to the vaccinated individuals. Overall, this leads to 48 differential equations and 48 variables. The optimal control formulation remains the same except that the state trajectories are now provided by the new system of ODEs.

Figure S1 shows the state diagram augmented to allow for breakthrough infection i.e. vaccinated individuals becoming infected. This is done by duplicating the disease progression states to allow for the decreased rates of infection, symptoms, hospitalization, and death in vaccinated individuals (see Table 3).

Recovery also provides some degree of immunity from COVID-19, though like vaccination the immunity is not foolproof [9]. To allow for reinfection (i.e. recovered individuals becoming infected), we consider that recovered individuals may become exposed, symptomatic, hospitalized, and deceased at decreased rates by making another copy of the disease states. The set of additional states can model differences in the level of protection afforded via vaccination versus that of acquired immunity.

Both breakthrough infections and reinfection introduce multiplicative factors that represent the decreased rate of exposure, symptoms, hospitalization, and death for vaccinated and recovered individuals [12, 9]. Note that the factors need not be the same for vaccinated and recovered. We now provide the notation and typical values for the respective decreases in the table below along with the relevant sources.

This generalization was used throughout all numerical results presented in Section 4.

2.2 Multi-dose vaccination

We also generalize our model to allow for two dose vaccinations. Results for our multi-dose model can be found in Section 4.8. For simplicity we illustrate for the case that an individual does not contract the disease either after recovery or after receiving both doses. These assumptions can be relaxed by expanding the state space as in Section 2.1.

We now describe how the ODE formulation for one dose vaccines can be adapted to represent the dynamics when vaccines have two doses. We append to our model an intermediate vaccination state as in Figure S2 in which individuals can be infected, develop symptoms, are hospitalized, and die with

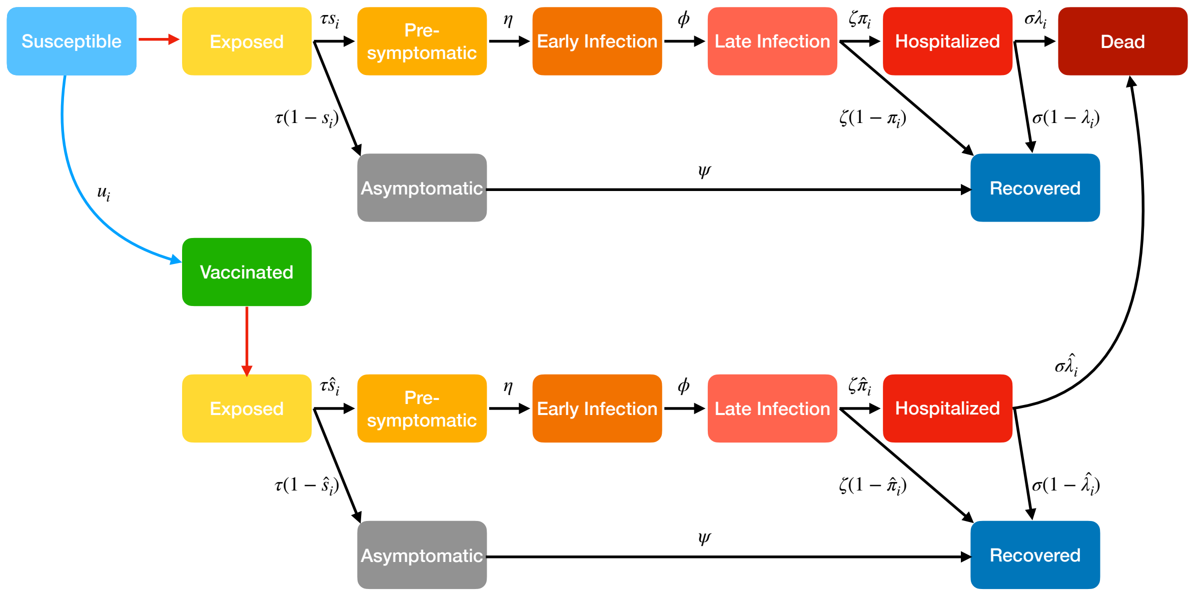

Figure S1: The above diagram depicts the generalized disease states in the single dose vaccine model when expanded to allow for breakthrough infections.

Table 3: Vaccine Parameters

	Description	Value(s)	Ref.
v_e	Multiplicative factor in exposure rate due to vaccination	0.5	[12],[13]
v_s	Multiplicative factor in symptomatic rate due to vaccination	0.3	[12],[13]
v_h	Multiplicative factor in hospitalization rate due to vaccination	0.2	[12],[13]
v_d	Multiplicative factor in death rate due to vaccination	0.1	[12],[13]
r_e	Multiplicative factor in exposure rate due to reinfection	0.5	[9]
r_s	Multiplicative factor in symptomatic rate due to reinfection	0.3	[9]
r_h	Multiplicative factor in hospitalization rate due to reinfection	0.2	[9]
r_d	Multiplicative factor in death rate due to reinfection	0.1	[9]

lower probability than susceptibles [14, 12, 13]. Susceptibles transition to this state after receiving one dose. After individuals receive both doses they transition from this state to the fully vaccinated state. Let $u_i^*(t)$ refer to the vaccination rate for the second dose of group i. This is reflected in similar augmentation to the system of ODEs in Figure 2. We refer to the set of state trajectories for this appended 2 dose system as $\mathcal{S}'$.

Obtaining the optimal vaccination strategy requires the solution of an additional decision problem: the order of the second dose among different groups. For example, should the first dose be administered to as many people as possible first and administering the second dose ought to start only after everyone had received the first dose? Or should an individual be administered the second dose right after the minimum mandated period from the first dose, while others await their first dose? There can be several combinations of the above extremes, eg, depending on which group they belong to, some individuals ought to wait for their second dose until everyone receives the first dose, others receive their second dose as soon as possible after the first dose. We describe the optimal control formulation that will determine the optimal rates for administering first and second doses for different groups and times subject to the capacity constraint for vaccine delivery.

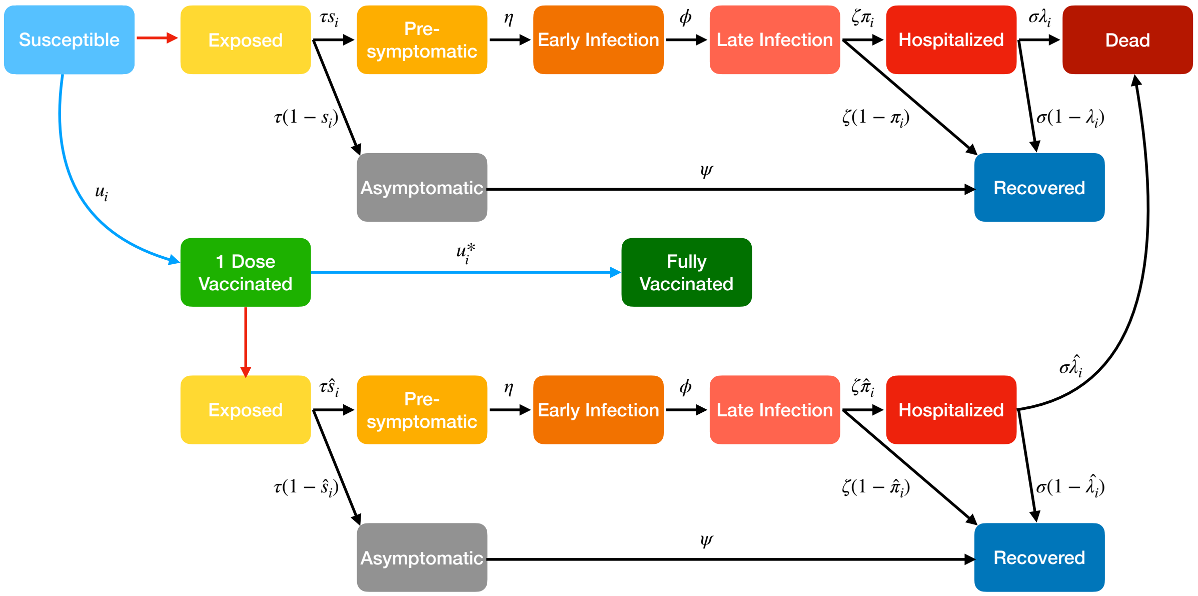

Figure S2: In this expanded two dose state diagram, we introduce the interim "1 Dose Vaccinated" state in which infection is possible but with lower rates as well as decreased risk of symptoms, hospitalization, and death (denoted by $\hat{s}_i < s_i$, $\hat{\pi}_i < \pi_i$, and $\hat{\lambda}_i < \lambda_i$, respectively). Again, for ease of depiction, we present the simplified case in which fully vaccinated and recovered individuals cannot become infected.

$$
\begin{aligned}
\text{minimize} \quad & \sum_{i\in\{X,Y,Z\}} D_i(T) \\
\text{subject to } & x \in \mathcal{S}' \\
& x(0) = x_0, \\
& 0 \le u_i(t), u_i^*(t) \le 1 \quad \forall i \in \{X,Y,Z\}, t \in [0,T] \\
& U^*(t) \le V_0 \quad \forall t \in [0,T]
\end{aligned} \tag{1}
$$

where $U^*(t) = \sum_{i\in\{X,Y,Z\}} S_i u_i + V_i u_i^*$ is the fraction of individuals vaccinated at a given time.

The optimal vaccination strategy can be obtained by solving the above optimal control formulations using the numerical tools described in Section 3.

In 2 dose vaccination, **we define parameter α that denotes the partial protection (as fraction of two dose protection levels in Table 3) afforded by one dose of the vaccine**. That is, the multiplicative factor on exposure after 1 dose will be $1-\alpha(1-v_e)$. We vary α between 0.4 and 0.8 in line with clinical estimates [15]. Findings from our numerical computations using this model are included in Section 4.8.

2.3 Different objective functions

Our computational framework is also flexible enough to cater to different public health objectives as shown in Section 4.7. Public health objectives other than the total death count can be minimized by appropriately replacing the objective function. One can minimize the time average of the hospitalization count, total years of life lost (YLL), time average of the symptomatic count, or some combination of these or other metrics of interest. This is noteworthy as an increasing body of work seeks to grapple with the socioeconomic costs of pandemics or even the impacts on social justice [16, 17, 18]. To minimize the time average of the hospitalization count, the objective function in the optimal control formulation in

1 needs to be $\frac{\sum_{i\in\{X,Y,Z\}}\int_0^T H_i(t)dt}{T}$. From the ODEs in Figure 2, $D_i(T) = \sigma\lambda_i \int_0^T H_i(t)dt$. The objective function can therefore be expressed as a weighted sum of the cumulative death count in each group: $\sum_{i\in\{X,Y,Z\}} \frac{D_i(T)}{T\sigma\lambda_i}$. YLLs are computed as a weighted sum of death counts over the different groups where the count in each group is weighted according to the difference between overall life expectancy and the group's mean age (the weight is set to 0 if the difference turns out to be negative). To minimize the time average of the symptomatic count, the objective function has to be $\frac{\sum_{i\in\{X,Y,Z\}}\int_0^T (I_i(t)+L_i(t))dt}{T}$. The time average of the hospitalization counts and symptomatic counts represent number of hospitalized and symptomatic individuals per day respectively; these also respectively represent the number of man days lost due to hospitalization and symptoms normalized by the size of the interval under consideration. The framework as a whole and the optimal control formulation remains the same otherwise.

References

[1] *Coronavirus disease (covid-19): How is it transmitted?* URL: https://www.who.int/news-room/questions-and-answers/item/coronavirus-disease-covid-19-how-is-it-transmitted.

[2] *Covid-19 pandemic planning scenarios.* URL: https://www.cdc.gov/coronavirus/2019-ncov/hcp/planning-scenarios.html.

[3] *Risk for COVID-19 infection, hospitalization, and death by age group.* URL: https://www.cdc.gov/coronavirus/2019-ncov/covid-data/investigations-discovery/hospitalization-death-by-age.html.

[4] *Presymptomatic transmission of SARS-COV-2 - Singapore, January 23–March 16, 2020.* Apr. 2020. URL: https://www.cdc.gov/mmwr/volumes/69/wr/mm6914e1.htm#:~:text=Presymptomatic%5C%20tran.

[5] Jennifer K Bender et al. "Analysis of asymptomatic and presymptomatic transmission in SARS-CoV-2 outbreak, Germany, 2020". In: *Emerging infectious diseases* 27.4 (2021), p. 1159.

[6] *Contact tracing for covid-19.* URL: https://www.cdc.gov/coronavirus/2019-ncov/php/contact-tracing/contact-tracing-plan/contact-tracing.html.

[7] *Interim clinical guidance for management of patients with confirmed coronavirus disease (covid-19).* URL: https://stacks.cdc.gov/view/cdc/88624.

[8] *Mortality analyses.* URL: https://coronavirus.jhu.edu/data/mortality.

[9] *CDC COVID-19 study shows mrna vaccines reduce risk of infection by 91 percent for fully vaccinated people.* June 2021. URL: https://www.cdc.gov/media/releases/2021/p0607-mrna-reduce-risks.html.

[10] National Center for Immunization and Respiratory Diseases. "Science Brief: SARS-CoV-2 Infection-induced and Vaccine-induced Immunity". In: *CDC COVID-19 Science Briefs [Internet]*. Centers for Disease Control and Prevention (US), 2021.

[11] *Comparing the differences between covid-19 vaccines.* URL: https://www.mayoclinic.org/coronavirus-covid-19/vaccine/comparing-vaccines.

[12] Laith J Abu-Raddad, Hiam Chemaitelly, and Adeel A Butt. "Effectiveness of the BNT162b2 Covid-19 Vaccine against the B. 1.1. 7 and B. 1.351 Variants". In: *New England Journal of Medicine* 385.2 (2021), pp. 187–189.

[13] Srinivas Nanduri et al. "Effectiveness of Pfizer-BioNTech and Moderna vaccines in preventing SARS-CoV-2 infection among nursing home residents before and during widespread circulation of the SARS-CoV-2 B. 1.617. 2 (Delta) variant—National Healthcare Safety Network, March 1–August 1, 2021". In: *Morbidity and Mortality Weekly Report* 70.34 (2021), p. 1163.

[14] Victoria Hall et al. "Protection against SARS-CoV-2 after Covid-19 vaccination and previous infection". In: *New England Journal of Medicine* 386.13 (2022), pp. 1207–1220.

[15] Jamie Lopez Bernal et al. "Effectiveness of Covid-19 vaccines against the B. 1.617. 2 (Delta) variant". In: *New England Journal of Medicine* 385.7 (2021), pp. 585–594.

[16] Nicola Mulberryid et al. "Vaccine rollout strategies: The case for vaccinating essential workers early". In: *PLOS Global Public Health* 1 (10 Oct. 2021), e0000020. ISSN: 2767-3375. DOI: 10.1371/JOURNAL.PGPH.0000020. URL: https://journals.plos.org/globalpublichealth/article?id=10.1371/journal.pgph.0000020.

[17] Diego S Silva and Maxwell J Smith. "Social distancing, social justice, and risk during the COVID-19 pandemic". In: *Canadian journal of public health* 111 (2020), pp. 459–461.

[18] Lisa R Fortuna et al. "Inequity and the disproportionate impact of COVID-19 on communities of color in the United States: The need for a trauma-informed social justice response." In: *Psychological Trauma: Theory, Research, Practice, and Policy* 12.5 (2020), p. 443.

[19] Lev Semenovich Pontryagin. *Mathematical theory of optimal processes*. CRC press, 1987.

[20] Dieter Grass et al. *Optimal control of nonlinear processes with applications in drugs,corruption, and terror*. Springer, 2010.

[21] O. Wahltinez et al. "COVID-19 Open-Data: curating a fine-grained, global-scale data repository for SARS-CoV-2". In: (2020). Work in progress. URL: https://goo.gle/covid-19-open-data.

[22] *Report COVID-19: Essential Workers in the States*. URL: https://www.ncsl.org/labor-and-employment/covid-19-essential-workers-in-the-states.

[23] *US states with the most essential workers*. Dec. 2021. URL: https://unitedwaynca.org/blog/us-states-with-the-most-ssential-workers/.

[24] URL: https://bbs.portal.gov.bd/sites/default/files/files/bbs.portal.gov.bd/page/057b0f3b_a9e8_4fde_b3a6_6daec3853586/2021-12-02-10-01-a5b3adcd2ea20db89d4bae0c90bd86cf.pdf.

[25] *Population, total*. URL: https://data.worldbank.org/indicator/SP.POP.TOTL.

[26] Kiesha Prem, Alex R Cook, and Mark Jit. "Projecting social contact matrices in 152 countries using contact surveys and demographic data". In: *PLoS computational biology* 13.9 (2017), e1005697.

[27] Kate M Bubar et al. "Model-informed COVID-19 vaccine prioritization strategies by age and serostatus". In: *Science* 371.6532 (2021), pp. 916–921.

[28] Edouard Mathieu et al. "A global database of COVID-19 vaccinations". In: *Nature human behaviour* 5.7 (2021), pp. 947–953.

[29] Claire Klobucista. *By how much are countries underreporting COVID-19 cases and deaths?* URL: https://www.cfr.org/in-brief/how-much-are-countries-underreporting-covid-19-cases-and-deaths.

[30] Yusha Araf et al. "Omicron variant of SARS-CoV-2: genomics, transmissibility, and responses to current COVID-19 vaccines". In: *Journal of medical virology* 94.5 (2022), pp. 1825–1832.

[31] Kathy Katella. *Omicron, Delta, Alpha, and more: What to know about the coronavirusnbsp;variants*. Feb. 2023. URL: https://www.yalemedicine.org/news/covid-19-variants-of-concern-omicron.

[32] Joe Hilton and Matt J Keeling. "Estimation of country-level basic reproductive ratios for novel Coronavirus (SARS-CoV-2/COVID-19) using synthetic contact matrices". In: *PLoS computational biology* 16.7 (2020), e1008031.

[33] Nadya Johanna, Henrico Citrawijaya, and Grace Wangge. "Mass screening vs lockdown vs combination of both to control COVID-19: A systematic review". In: *Journal of public health research* 9.4 (2020), jphr–2020.

[34] Celine McNicholas and Margaret Poydock. *Who are essential workers?: A comprehensive look at their wages, demographics, and unionization rates*. May 2020. URL: https://www.epi.org/blog/who-are-essential-workers-a-comprehensive-look-at-their-wages-demographics-and-unionization-rates/.

[35] J O'grady et al. *Tuberculosis in prisons: anatomy of global neglect*. 2011.

[36] *Federal Bureau of Prisons*. URL: https://www.bop.gov/about/statistics/population_statistics.jsp.

[37] *FASTSTATS - Residential Care Community*. Dec. 2022. URL: https://www.cdc.gov/nchs/fastats/residential-care-communities.htm.

[38] Martial L Ndeffo-Mbah et al. "Dynamic models of infectious disease transmission in prisons and the general population". In: *Epidemiologic reviews* 40.1 (2018), pp. 40–57.

[39] Andrew T Levin et al. "COVID-19 prevalence and mortality in longer-term care facilities". In: *European Journal of Epidemiology* (2022), pp. 1–8.

[40] Courtney H Van Houtven, Nathan A Boucher, and Walter D Dawson. "Impact of the COVID-19 outbreak on long-term care in the United States". In: *International Long-Term Care Policy Network* (2020).

[41] Jack H Buckner, Gerardo Chowell, and Michael R Springborn. "Dynamic prioritization of COVID-19 vaccines when social distancing is limited for essential workers". In: *Proceedings of the National Academy of Sciences* 118.16 (2021).

[42] Rajan Patel, Ira M Longini Jr, and M Elizabeth Halloran. "Finding optimal vaccination strategies for pandemic influenza using genetic algorithms". In: *Journal of theoretical biology* 234.2 (2005), pp. 201–212.

[43] URL: https://www.cdc.gov/covid/hcp/clinical-care/underlying-conditions.html#cdc_generic_section_6-key-findings-from-one-large-cross-sectional-study.

[44] Oct. 2022. URL: https://www.cdc.gov/nchs/products/databriefs/db446.htm.

[45] Jan. 2024. URL: https://www.ssa.gov/pubs/EN-05-10043.pdf.

[46] URL: https://www.census.gov/popclock/.

[47] Dana Braga and Richard Fry. *1. the growth of the older workforce*. Dec. 2023. URL: https://www.pewresearch.org/social-trends/2023/12/14/the-growth-of-the-older-workforce/#:~:text=Some%2019%25%20of%20adults%20ages,18%25%20of%20older%20Americans%20worked..

[48] Jr. John J. DiIulio et al. *Public service and the Federal Government*. June 2023. URL: https://www.brookings.edu/articles/public-service-and-the-federal-government/#:~:text=and%20small%20businesses.-,Across%20the%20U.S.%2C%20nearly%2024%20million%20people%E2%80%94a%20little%20over,in%20state%20and%20local%20governments..

[49] URL: https://www.bls.gov/careeroutlook/2017/article/older-workers.htm.

[50] Samuel Stebbins, Grant Suneson, and Douglas A. McIntyre. *These are the jobs with the oldest workforces in the United States, from farmers to shuttle drivers*. Oct. 2021. URL: https://www.usatoday.com/story/news/nation/2021/10/26/these-jobs-have-oldest-workforce-country/6166671001/.

[51] URL: https://www.lung.org/lung-health-diseases/lung-disease-lookup/asthma/learn-about-asthma/types/severe-asthma#:~:text=Diagnosing%20Severe%20Asthma&text=Of%20the%20more%20than%2025,or%20are%20just%20uncontrolled%20asthma..

[52] Eileen Wang et al. "Characterization of severe asthma worldwide: data from the International Severe Asthma Registry". In: *Chest* 157.4 (2020), pp. 790–804.

[53] URL: https://www.cancer.org/cancer/managing-cancer/side-effects/infections/preventing-infections-in-people-with-cancer.html.

[54] Eric S Donkor. "Stroke in the 21st century: a snapshot of the burden, epidemiology, and quality of life". In: *Stroke research and treatment* 2018.1 (2018), p. 3238165.

[55] Mohammed Yousufuddin and Nathan Young. "Aging and ischemic stroke". In: *Aging (Albany NY)* 11.9 (2019), p. 2542.

[56] URL: https://esrdnetworks.org/resources-news/national-esrd-census-data/#:~:text=National%20ESRD%20Data%20as%20of,%5Badd%20access%20date%20here%5D..

[57] Centers for Disease Control, Prevention, et al. "Chronic kidney disease in the United States, 2023". In: *Atlanta, GA: US Department of Health and Human Services, Centers for Disease Control and Prevention* (2023).

[58] URL: https://www.lung.org/about-us/our-impact#:~:text=More%20than%2035%20million%20people,living%20with%20a%20lung%20disease..

[59] Oct. 2024. URL: https://aafa.org/asthma/asthma-facts/#:~:text=Asthma%20can%20be%20deadly%20if,of%20Asthma%20Attacks%20in%20Children?.

[60] Hope Gillette. *Working with COPD: What are the options?* Jan. 2023. URL: https://www.healthline.com/health/can-you-work-with-copd.

[61] Nov. 2023. URL: https://www.cdc.gov/nchs/fastats/liver-disease.htm#:~:text=Number%20of%20adults%20age%2018,with%20diagnosed%20liver%20disease:%201.8%25.

[62] Daniela P Ladner et al. "Increasing prevalence of cirrhosis among insured adults in the United States, 2012–2018". In: *PloS one* 19.2 (2024), e0298887.

[63] URL: https://www.cff.org/intro-cf/about-cystic-fibrosis#:~:text=the%20United%20States:-,There%20are%20close%20to%2040%2C000%20children%20and%20adults%20living%20with,is%20age%2018%20or%20older..

[64] Anjali D Deshpande, Marcie Harris-Hayes, and Mario Schootman. "Epidemiology of diabetes and diabetes-related complications". In: *Physical therapy* 88.11 (2008), pp. 1254–1264.

[65] Taylor M Shockey, Rebecca J Tsai, and Pyone Cho. "Prevalence of diagnosed diabetes among employed us adults by demographic characteristics and occupation, 36 states, 2014 to 2018". In: *Journal of occupational and environmental medicine* 63.4 (2021), pp. 302–310.

[66] Anna Milanesi and Jane E Weinreb. "Diabetes in the elderly". In: (2015).

[67] Biykem Bozkurt et al. "Heart failure epidemiology and outcomes statistics: a report of the Heart Failure Society of America". In: (2023).

[68] Michael W Rich. "Heart failure in the 21st century: a cardiogeriatric syndrome". In: *The Journals of Gerontology Series A: Biological Sciences and Medical Sciences* 56.2 (2001), pp. M88–M96.

[69] URL: https://www.hiv.gov/hiv-basics/overview/data-and-trends/statistics#:~:text=At%20year%2Dend%202022%2C%20an,to%20the%20latest%20CDC%20data:.

[70] URL: https://www.hiv.gov/hiv-basics/living-well-with-hiv/taking-care-of-yourself/aging-with-hiv#:.

[71] Samuel D Emmerich et al. "Obesity and Severe Obesity Prevalence in Adults: United States, August 2021–August 2023". In: (2024).

[72] Yizhe Lim and Joshua Boster. "Obesity and comorbid conditions". In: (2021).

[73] URL: https://www.niaid.nih.gov/diseases-conditions/primary-immune-deficiency-diseases-pidds.

[74] URL: https://www.cdc.gov/primary-immunodeficiency/about/index.html#:~:text=Washing%20your%20hands%20the%20right,prior%20to%20treatment%20for%20SCID..

[75] Saramoriarty. *U.S. reaches historic milestone of 1 million transplants*. Oct. 2023. URL: https://unos.org/news/u-s-reaches-1-million-transplants/#:~:text=More%20than%20400%2C000%20people%20are%20alive%20today%20with%20a%20functioning%20transplant..

[76] URL: https://www.kidney.org.uk/are-work-and-a-normal-life-possible-after-a-transplant#:~:text=It%20is%20usual%20to%20take,directly%20on%20to%20the%20kidney..

[77] Paula M Williams. "Tuberculosis—United States, 2023". In: *MMWR. Morbidity and Mortality Weekly Report* 73 (2024).

[78] Aug. 2024. URL: https://www.dhs.wisconsin.gov/tb/precautions.htm#:~:text=Patients%20with%20confirmed%20infectious%20TB,is%20deemed%20to%20be%20noninfectious..

[79] Oct. 2024. URL: https://www.kff.org/other/state-indicator/distribution-by-age/?currentTimeframe=0&sortModel=%7B%22colId%22%3A%22Location%22%2C%22sort%22%3A%22asc%22%7D.
