# Supplementary material for: Protect or prevent? A practicable framework for the dilemmas of COVID-19 vaccine prioritization
Source: PLoS One. 2025 Jan 22;20(1):e0316294. doi: 10.1371/journal.pone.0316294 (PMC11753641; doi:10.1371/journal.pone.0316294)
Supplement: S3 Appendix — (PDF) [file pone.0316294.s003.pdf]

Protect or prevent? A practicable framework for the dilemmas of COVID-19 vaccine prioritization
Supporting Information

Raghu Arghal[1*], Harvey Rubin[2], Shirin Saeedi Bidokhti[1], Saswati Sarkar[1]

March 2023

1 Department of Electrical and Systems Engineering, University of Pennsylvania, Philadelphia, PA, United States

2 Division of Infectious Diseases, Department of Medicine, University of Pennsylvania School of Medicine, Philadelphia, PA, United States

***** Corresponding Author (rarghal@seas.upenn@edu)

3 Proof of Theorem 4.1

We now restate and prove Theorem 4.1 from section 4.2. For a policy $u(t)$, we will use $U(t)$ to denote the vaccine allocation in absolute quantity rather than fraction i.e. $U_i(t) = u_i(t)S_i(t)$.

Theorem 4.1
There exists an optimal vaccination strategy u such that, for each t there exist $a(t), b(t), c(t) \in \{X, Y, Z\}$ all distinct and $U_{a(t)}(t) = min\left(V_0, S_{a(t)}(t)\right)$, $U_{b(t)}(t) = min\left(S_{b(t)}(t), V_0 - U_{a(t)}(t)\right)$, and $U_{c(t)}(t) = min\left(S_{c(t)}(t), V_0 - U_{a(t)}(t) - U_{b(t)}(t)\right)$.

Here we present a proof of Theorem 4.1 in the simplified system which does not allow for reinfection or breakthrough infection. After formulating vaccine prioritization as an optimal control problem, we use Pontryagin's Maximum Principle to obtain necessary conditions for an optimal control from which we discern structural properties [19], [20].

We first formulate the Hamiltonian and Lagrangian as follows:

$$\mathcal{H} := \sum_{i\in\{X,Y,Z\}} (\lambda_i^S \dot{S}_i + \lambda_i^E \dot{E}_i + \lambda_i^P \dot{P}_i + \lambda_i^a \dot{A}_i + \lambda_i^I \dot{I}_i + \lambda_i^L \dot{L}_i + \lambda_i^H \dot{H}_i) \tag{2}$$

$$\mathcal{L} := \mathcal{H} - \mu \left(\sum_{i\in\{X,Y,Z\}} S_i u_i - V_0 \right) \tag{3}$$

where the λ costate functions are absolutely continuous and satisfy

$$\dot{\lambda}_i^S = -\frac{\partial \mathcal{L}}{\partial S_i} \quad \dot{\lambda}_i^E = -\frac{\partial \mathcal{L}}{\partial E_i} \quad \dot{\lambda}_i^P = -\frac{\partial \mathcal{L}}{\partial P_i} \quad \dot{\lambda}_i^A = -\frac{\partial \mathcal{L}}{\partial A_i} \quad \dot{\lambda}_i^I = -\frac{\partial \mathcal{L}}{\partial I_i} \quad \dot{\lambda}_i^L = -\frac{\partial \mathcal{L}}{\partial L_i} \quad \dot{\lambda}_i^H = -\frac{\partial \mathcal{L}}{\partial H_i} \tag{4}$$

$$0 = \lambda_i^S(T) \quad 0 = \lambda_i^E(T) \quad 0 = \lambda_i^P(T) \quad 0 = \lambda_i^A(T) \quad 0 = \lambda_i^I(T) \quad 0 = \lambda_i^L(T) \quad 0 = \lambda_i^H(T) \tag{5}$$

and μ is an integrable function satisfying

$$\mu \left(\sum_{i\in\{X,Y,Z\}} S_i u_i - V_0 \right) = 0, \quad \mu(t) \geq 0 \ a.e. \tag{6}$$

$$n_i(t) = -\lambda_i^S S_i - \mu S_i \ a.e. \text{ where } n_i(t) \in N_{[0,1]}(u_i^*(t)) \tag{7}$$

where $N_{[0,1]}$ denotes the normal cone to $[0, 1]$.

PMP then states that $u^* \in \text{argmax } \mathcal{H}$ where u^* refers to an optimal control function. Note that the control variable is only included in the $\dot{S}_i$ term of the Hamiltonian. Thus, maximizing the Hamiltonian can be reduced to the following:

$$u^*(t) \in \text{argmax}_{u\in\mathcal{U}} \sum_{i\in\{X,Y,Z\}} -\lambda_i^S S_i u_i \tag{8}$$

where $\mathcal{U} = \{v : 0 \preceq v \preceq 1, \sum_{i\in\{X,Y,Z\}} S_i v_i \leq V_0\}$ denotes the set of admissible controls.
Let $a(t), b(t), c(t) \in \{X, Y, Z\}$ be distinct such that $-\lambda_{a(t)}^S S_{a(t)} \geq -\lambda_{b(t)}^S S_{b(t)} \geq -\lambda_{c(t)}^S S_{c(t)}$. Subject to choosing a control in $\mathcal{U}$, (8) implies that $u_{a(t)}^*(t)$ be maximized, subject to the same $u_{b(t)}^*(t)$ be maximized and so on. We now elaborate three cases.

1. Case 1: $S_{a(t)} \geq V_0$
   In this case, there are enough susceptible individuals in the highest priority group to fully consume vaccine capacity. Thus, $u_{a(t)}^*(t) = \frac{V_0}{S_{a(t)}}$ and $u_{b(t)}^*(t) = u_{c(t)}^*(t) = 0$.

2. Case 2: $S_{a(t)} < V_0 \leq S_{a(t)} + S_{b(t)}$
   Here the vaccine capacity exceeds the size of the susceptible population in the highest priority group and excess capacity is passed to the second priority group. Thus, $u^*_{a(t)}(t) = 1$, $u^*_{b(t)}(t) = \frac{V_0 - S_{a(t)}}{S_{b(t)}}$, and $u^*_{c(t)}(t) = 0$.

3. Case 3: $S_{a(t)} + S_{b(t)} < V_0$
   In this case, the vaccine capacity exceeds the total susceptible population of the first two priority groups, and any further vaccines are allocated to the final group until no further susceptible individuals remain. Thus, $u^*_{a(t)}(t) = u^*_{b(t)}(t) = 1$ and $u^*_{c(t)}(t) = \min\left(\frac{V_0 - S_{a(t)} - S_{b(t)}}{S_{c(t)}}, 1\right)$

This directly leads to Theorem 4.1 considering that $U_i(t) = u_i(t)S_i(t)$ for each i, t.

References

[1] *Coronavirus disease (covid-19): How is it transmitted?* URL: https://www.who.int/news-room/questions-and-answers/item/coronavirus-disease-covid-19-how-is-it-transmitted.

[2] *Covid-19 pandemic planning scenarios.* URL: https://www.cdc.gov/coronavirus/2019-ncov/hcp/planning-scenarios.html.

[3] *Risk for COVID-19 infection, hospitalization, and death by age group.* URL: https://www.cdc.gov/coronavirus/2019-ncov/covid-data/investigations-discovery/hospitalization-death-by-age.html.

[4] *Presymptomatic transmission of SARS-COV-2 - Singapore, January 23–March 16, 2020.* Apr. 2020. URL: https://www.cdc.gov/mmwr/volumes/69/wr/mm6914e1.htm#:~:text=Presymptomatic%5C%20tran.

[5] Jennifer K Bender et al. "Analysis of asymptomatic and presymptomatic transmission in SARS-CoV-2 outbreak, Germany, 2020". In: *Emerging infectious diseases* 27.4 (2021), p. 1159.

[6] *Contact tracing for covid-19.* URL: https://www.cdc.gov/coronavirus/2019-ncov/php/contact-tracing/contact-tracing-plan/contact-tracing.html.

[7] *Interim clinical guidance for management of patients with confirmed coronavirus disease (covid-19).* URL: https://stacks.cdc.gov/view/cdc/88624.

[8] *Mortality analyses.* URL: https://coronavirus.jhu.edu/data/mortality.

[9] *CDC COVID-19 study shows mrna vaccines reduce risk of infection by 91 percent for fully vaccinated people.* June 2021. URL: https://www.cdc.gov/media/releases/2021/p0607-mrna-reduce-risks.html.

[10] National Center for Immunization and Respiratory Diseases. "Science Brief: SARS-CoV-2 Infection-induced and Vaccine-induced Immunity". In: *CDC COVID-19 Science Briefs [Internet]*. Centers for Disease Control and Prevention (US), 2021.

[11] *Comparing the differences between covid-19 vaccines.* URL: https://www.mayoclinic.org/coronavirus-covid-19/vaccine/comparing-vaccines.

[12] Laith J Abu-Raddad, Hiam Chemaitelly, and Adeel A Butt. "Effectiveness of the BNT162b2 Covid-19 Vaccine against the B. 1.1. 7 and B. 1.351 Variants". In: *New England Journal of Medicine* 385.2 (2021), pp. 187–189.

[13] Srinivas Nanduri et al. "Effectiveness of Pfizer-BioNTech and Moderna vaccines in preventing SARS-CoV-2 infection among nursing home residents before and during widespread circulation of the SARS-CoV-2 B. 1.617. 2 (Delta) variant—National Healthcare Safety Network, March 1–August 1, 2021". In: *Morbidity and Mortality Weekly Report* 70.34 (2021), p. 1163.

[14] Victoria Hall et al. "Protection against SARS-CoV-2 after Covid-19 vaccination and previous infection". In: *New England Journal of Medicine* 386.13 (2022), pp. 1207–1220.

[15] Jamie Lopez Bernal et al. "Effectiveness of Covid-19 vaccines against the B. 1.617. 2 (Delta) variant". In: *New England Journal of Medicine* 385.7 (2021), pp. 585–594.

[16] Nicola Mulberryid et al. "Vaccine rollout strategies: The case for vaccinating essential workers early". In: *PLOS Global Public Health* 1 (10 Oct. 2021), e0000020. ISSN: 2767-3375. DOI: 10.1371/JOURNAL.PGPH.0000020. URL: https://journals.plos.org/globalpublichealth/article?id=10.1371/journal.pgph.0000020.

[17] Diego S Silva and Maxwell J Smith. "Social distancing, social justice, and risk during the COVID-19 pandemic". In: *Canadian journal of public health* 111 (2020), pp. 459–461.

[18] Lisa R Fortuna et al. "Inequity and the disproportionate impact of COVID-19 on communities of color in the United States: The need for a trauma-informed social justice response." In: *Psychological Trauma: Theory, Research, Practice, and Policy* 12.5 (2020), p. 443.

[19] Lev Semenovich Pontryagin. *Mathematical theory of optimal processes*. CRC press, 1987.

[20] Dieter Grass et al. *Optimal control of nonlinear processes with applications in drugs,corruption, and terror*. Springer, 2010.

[21] O. Wahltinez et al. "COVID-19 Open-Data: curating a fine-grained, global-scale data repository for SARS-CoV-2". In: (2020). Work in progress. URL: https://goo.gle/covid-19-open-data.

[22] *Report COVID-19: Essential Workers in the States*. URL: https://www.ncsl.org/labor-and-employment/covid-19-essential-workers-in-the-states.

[23] *US states with the most essential workers*. Dec. 2021. URL: https://unitedwaynca.org/blog/us-states-with-the-most-ssential-workers/.

[24] URL: https://bbs.portal.gov.bd/sites/default/files/files/bbs.portal.gov.bd/page/057b0f3b_a9e8_4fde_b3a6_6daec3853586/2021-12-02-10-01-a5b3adcd2ea20db89d4bae0c90bd86cf.pdf.

[25] *Population, total*. URL: https://data.worldbank.org/indicator/SP.POP.TOTL.

[26] Kiesha Prem, Alex R Cook, and Mark Jit. "Projecting social contact matrices in 152 countries using contact surveys and demographic data". In: *PLoS computational biology* 13.9 (2017), e1005697.

[27] Kate M Bubar et al. "Model-informed COVID-19 vaccine prioritization strategies by age and serostatus". In: *Science* 371.6532 (2021), pp. 916–921.

[28] Edouard Mathieu et al. "A global database of COVID-19 vaccinations". In: *Nature human behaviour* 5.7 (2021), pp. 947–953.

[29] Claire Klobucista. *By how much are countries underreporting COVID-19 cases and deaths?* URL: https://www.cfr.org/in-brief/how-much-are-countries-underreporting-covid-19-cases-and-deaths.

[30] Yusha Araf et al. "Omicron variant of SARS-CoV-2: genomics, transmissibility, and responses to current COVID-19 vaccines". In: *Journal of medical virology* 94.5 (2022), pp. 1825–1832.

[31] Kathy Katella. *Omicron, Delta, Alpha, and more: What to know about the coronavirusnbsp;variants*. Feb. 2023. URL: https://www.yalemedicine.org/news/covid-19-variants-of-concern-omicron.

[32] Joe Hilton and Matt J Keeling. "Estimation of country-level basic reproductive ratios for novel Coronavirus (SARS-CoV-2/COVID-19) using synthetic contact matrices". In: *PLoS computational biology* 16.7 (2020), e1008031.

[33] Nadya Johanna, Henrico Citrawijaya, and Grace Wangge. "Mass screening vs lockdown vs combination of both to control COVID-19: A systematic review". In: *Journal of public health research* 9.4 (2020), jphr–2020.

[34] Celine McNicholas and Margaret Poydock. *Who are essential workers?: A comprehensive look at their wages, demographics, and unionization rates*. May 2020. URL: https://www.epi.org/blog/who-are-essential-workers-a-comprehensive-look-at-their-wages-demographics-and-unionization-rates/.

[35] J O'grady et al. *Tuberculosis in prisons: anatomy of global neglect*. 2011.

[36] *Federal Bureau of Prisons*. URL: https://www.bop.gov/about/statistics/population_statistics.jsp.

[37] *FASTSTATS - Residential Care Community*. Dec. 2022. URL: https://www.cdc.gov/nchs/fastats/residential-care-communities.htm.

[38] Martial L Ndeffo-Mbah et al. "Dynamic models of infectious disease transmission in prisons and the general population". In: *Epidemiologic reviews* 40.1 (2018), pp. 40–57.

[39] Andrew T Levin et al. "COVID-19 prevalence and mortality in longer-term care facilities". In: *European Journal of Epidemiology* (2022), pp. 1–8.

[40] Courtney H Van Houtven, Nathan A Boucher, and Walter D Dawson. "Impact of the COVID-19 outbreak on long-term care in the United States". In: *International Long-Term Care Policy Network* (2020).

[41] Jack H Buckner, Gerardo Chowell, and Michael R Springborn. "Dynamic prioritization of COVID-19 vaccines when social distancing is limited for essential workers". In: *Proceedings of the National Academy of Sciences* 118.16 (2021).

[42] Rajan Patel, Ira M Longini Jr, and M Elizabeth Halloran. "Finding optimal vaccination strategies for pandemic influenza using genetic algorithms". In: *Journal of theoretical biology* 234.2 (2005), pp. 201–212.

[43] URL: https://www.cdc.gov/covid/hcp/clinical-care/underlying-conditions.html#cdc_generic_section_6-key-findings-from-one-large-cross-sectional-study.

[44] Oct. 2022. URL: https://www.cdc.gov/nchs/products/databriefs/db446.htm.

[45] Jan. 2024. URL: https://www.ssa.gov/pubs/EN-05-10043.pdf.

[46] URL: https://www.census.gov/popclock/.

[47] Dana Braga and Richard Fry. *1. the growth of the older workforce*. Dec. 2023. URL: https://www.pewresearch.org/social-trends/2023/12/14/the-growth-of-the-older-workforce/#:~:text=Some%2019%25%20of%20adults%20ages,18%25%20of%20older%20Americans%20worked..

[48] Jr. John J. DiIulio et al. *Public service and the Federal Government*. June 2023. URL: https://www.brookings.edu/articles/public-service-and-the-federal-government/#:~:text=and%20small%20businesses.-,Across%20the%20U.S.%2C%20nearly%2024%20million%20people%E2%80%94a%20little%20over,in%20state%20and%20local%20governments..

[49] URL: https://www.bls.gov/careeroutlook/2017/article/older-workers.htm.

[50] Samuel Stebbins, Grant Suneson, and Douglas A. McIntyre. *These are the jobs with the oldest workforces in the United States, from farmers to shuttle drivers*. Oct. 2021. URL: https://www.usatoday.com/story/news/nation/2021/10/26/these-jobs-have-oldest-workforce-country/6166671001/.

[51] URL: https://www.lung.org/lung-health-diseases/lung-disease-lookup/asthma/learn-about-asthma/types/severe-asthma#:~:text=Diagnosing%20Severe%20Asthma&text=Of%20the%20more%20than%2025,or%20are%20just%20uncontrolled%20asthma..

[52] Eileen Wang et al. "Characterization of severe asthma worldwide: data from the International Severe Asthma Registry". In: *Chest* 157.4 (2020), pp. 790–804.

[53] URL: https://www.cancer.org/cancer/managing-cancer/side-effects/infections/preventing-infections-in-people-with-cancer.html.

[54] Eric S Donkor. "Stroke in the 21st century: a snapshot of the burden, epidemiology, and quality of life". In: *Stroke research and treatment* 2018.1 (2018), p. 3238165.

[55] Mohammed Yousufuddin and Nathan Young. "Aging and ischemic stroke". In: *Aging (Albany NY)* 11.9 (2019), p. 2542.

[56] URL: https://esrdnetworks.org/resources-news/national-esrd-census-data/#:~:text=National%20ESRD%20Data%20as%20of,%5Badd%20access%20date%20here%5D..

[57] Centers for Disease Control, Prevention, et al. "Chronic kidney disease in the United States, 2023". In: *Atlanta, GA: US Department of Health and Human Services, Centers for Disease Control and Prevention* (2023).

[58] URL: https://www.lung.org/about-us/our-impact#:~:text=More%20than%2035%20million%20people,living%20with%20a%20lung%20disease..

[59] Oct. 2024. URL: https://aafa.org/asthma/asthma-facts/#:~:text=Asthma%20can%20be%20deadly%20if,of%20Asthma%20Attacks%20in%20Children?.

[60] Hope Gillette. *Working with COPD: What are the options?* Jan. 2023. URL: https://www.healthline.com/health/can-you-work-with-copd.

[61] Nov. 2023. URL: https://www.cdc.gov/nchs/fastats/liver-disease.htm#:~:text=Number%20of%20adults%20age%2018,with%20diagnosed%20liver%20disease:%201.8%25.

[62] Daniela P Ladner et al. "Increasing prevalence of cirrhosis among insured adults in the United States, 2012–2018". In: *PloS one* 19.2 (2024), e0298887.

[63] URL: https://www.cff.org/intro-cf/about-cystic-fibrosis#:~:text=the%20United%20States:-,There%20are%20close%20to%2040%2C000%20children%20and%20adults%20living%20with,is%20age%2018%20or%20older..

[64] Anjali D Deshpande, Marcie Harris-Hayes, and Mario Schootman. "Epidemiology of diabetes and diabetes-related complications". In: *Physical therapy* 88.11 (2008), pp. 1254–1264.

[65] Taylor M Shockey, Rebecca J Tsai, and Pyone Cho. "Prevalence of diagnosed diabetes among employed us adults by demographic characteristics and occupation, 36 states, 2014 to 2018". In: *Journal of occupational and environmental medicine* 63.4 (2021), pp. 302–310.

[66] Anna Milanesi and Jane E Weinreb. "Diabetes in the elderly". In: (2015).

[67] Biykem Bozkurt et al. "Heart failure epidemiology and outcomes statistics: a report of the Heart Failure Society of America". In: (2023).

[68] Michael W Rich. "Heart failure in the 21st century: a cardiogeriatric syndrome". In: *The Journals of Gerontology Series A: Biological Sciences and Medical Sciences* 56.2 (2001), pp. M88–M96.

[69] URL: https://www.hiv.gov/hiv-basics/overview/data-and-trends/statistics#:~:text=At%20year%2Dend%202022%2C%20an,to%20the%20latest%20CDC%20data:.

[70] URL: https://www.hiv.gov/hiv-basics/living-well-with-hiv/taking-care-of-yourself/aging-with-hiv#:.

[71] Samuel D Emmerich et al. "Obesity and Severe Obesity Prevalence in Adults: United States, August 2021–August 2023". In: (2024).

[72] Yizhe Lim and Joshua Boster. "Obesity and comorbid conditions". In: (2021).

[73] URL: https://www.niaid.nih.gov/diseases-conditions/primary-immune-deficiency-diseases-pidds.

[74] URL: https://www.cdc.gov/primary-immunodeficiency/about/index.html#:~:text=Washing%20your%20hands%20the%20right,prior%20to%20treatment%20for%20SCID..

[75] Saramoriarty. *U.S. reaches historic milestone of 1 million transplants*. Oct. 2023. URL: https://unos.org/news/u-s-reaches-1-million-transplants/#:~:text=More%20than%20400%2C000%20people%20are%20alive%20today%20with%20a%20functioning%20transplant..

[76] URL: https://www.kidney.org.uk/are-work-and-a-normal-life-possible-after-a-transplant#:~:text=It%20is%20usual%20to%20take,directly%20on%20to%20the%20kidney..

[77] Paula M Williams. "Tuberculosis—United States, 2023". In: *MMWR. Morbidity and Mortality Weekly Report* 73 (2024).

[78] Aug. 2024. URL: https://www.dhs.wisconsin.gov/tb/precautions.htm#:~:text=Patients%20with%20confirmed%20infectious%20TB,is%20deemed%20to%20be%20noninfectious..

[79] Oct. 2024. URL: https://www.kff.org/other/state-indicator/distribution-by-age/?currentTimeframe=0&sortModel=%7B%22colId%22%3A%22Location%22%2C%22sort%22%3A%22asc%22%7D.
