# Supplementary material for: Protect or prevent? A practicable framework for the dilemmas of COVID-19 vaccine prioritization
Source: PLoS One. 2025 Jan 22;20(1):e0316294. doi: 10.1371/journal.pone.0316294 (PMC11753641; doi:10.1371/journal.pone.0316294)
Supplement: S4 Appendix — (PDF) [file pone.0316294.s004.pdf]

Protect or prevent? A practicable framework for the dilemmas of COVID-19 vaccine prioritization
Supporting Information

Raghu Arghal[1*], Harvey Rubin[2], Shirin Saeedi Bidokhti[1], Saswati Sarkar[1]

March 2023

1 Department of Electrical and Systems Engineering, University of Pennsylvania, Philadelphia, PA, United States

2 Division of Infectious Diseases, Department of Medicine, University of Pennsylvania School of Medicine, Philadelphia, PA, United States

***** Corresponding Author (rarghal@seas.upenn@edu)

4 Numerical evaluations

4.1 Model validation

We first describe how we obtain the parameters of our model for validation as described in Section 4.1. We consider publicly available data on infection and mortality counts in all US states and 139 countries [21]. Overall we considered a period extending from 04/01/2020 to 01/01/2021, the period between early reported deaths and the introduction of vaccines. We assume all initially infected individuals are in the baseline group. We obtain the initial values of the states from the publicly available infection counts on 04/01/2020 and the sizes of the different groups. We fix all individuals in age groups exceeding 65 as high risk. We obtain the fraction of the population in this group from publicly available population demographics [21]; this is the fraction of the population that is high risk. Percentage of workers characterized as essential workers[1] in US states is available in publicly available databases [23]. We use this fraction times the fraction of individuals of working age (20-65 years) as the fraction of the population that is high contact. Note that this estimate undercounts the fraction of high contact individuals as this does not consider unorganized private sector employees such as rideshare drivers who have high contact rates. For countries other than the US, we estimated the fraction of high contact individuals from economic data (percentage of GDP in service industries) and demographics (age-stratified population counts). That is, if $x\%$ of the country's economy was in the service industry (retail trade, transportation, and real estate being the largest share), then the high contact population was set to $x\%$ of people aged 20 to 65 [24]. We obtained disease parameters from CDC and WHO (Table 1, Supporting Information). After choosing the parameters as above, we determine the contact rate matrix using regression. The matrix consists of contact rates within each group (baseline, high risk, high contact) and across groups. These contact rates were selected to minimize mean squared normalized error (MMSNE) between the true infection and death counts in the respective area, and those projected for these locations via our model. We selected different sets of contact rates for different periods, each with a duration of two months (encompassing the time of investigation from 04/01/2020 to 01/01/2021). Different contact rates were chosen for different periods to account for changes in both government policy (e.g. start, relaxation, and end of lockdowns) and school openings which happen at low frequency.

Model Validation Outliers When regressing against actual infection and death counts [21], there were four countries (out of the total 139) that had outlying high MMSNE: Japan (JP), Lesotho (LS), Gambia (GM), and Mauritania (MR). While the max MMSNE of all other countries was 0.05, these four countries had MMSNE of 2.08, 10.16, 4.92, and 10.10, respectively. Here we seek to understand the factors that led to the poor fit. Each following subsection illustrates the fit of our model to the respective country's infection and death counts and posits feasible explanations for the high mismatch.

Japan is notable as the one country of the four outliers with a relatively high population size. In fact, Japan is the only of the four included in the top 125 countries by population [25]. As such, one would expect to see convergence between the system of ODEs and real data based on the Central Limit Theorem. However, the available case count data for Japan is flawed as the cumulative infection decreases over time which is impossible in practice. This artifact of the data collection did not allow us to fit real data. We therefore do not consider Japan.

Lesotho, with a population just over 2.2M, exhibits very low case counts and deaths [25]. As shown in Figure S3, this leads to non-smooth behavior in the real data which inhibits our ability to fit our model well.

Next, we consider Gambia. In addition to low overall counts, Gambia's data shows a sharp, sudden increase in death count as seen in Figure S4b. This is likely an artifact of delayed or imperfect record keeping of COVID-19 deaths and inhibits the fitting of smooth system dynamics.

Mauritania, though it has a small number of cases, does exhibit relatively smooth case and death curves. However, at the onset of our time window, Mauritania had very few confirmed cases. Thus,

[1] This group is composed of those working in critical industries who are not able to self isolate such as child care, energy, and transportation [22].

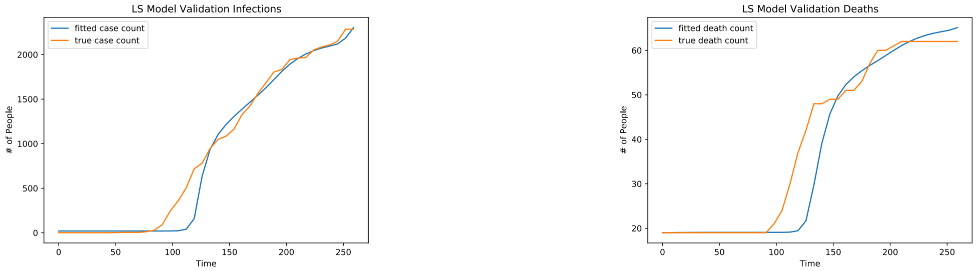

(a) Our model fit to Lesotho's infection counts (b) Our model fit to Lesotho's death counts

Figure S3: Lesotho COVID-19 Dynamics Fitting

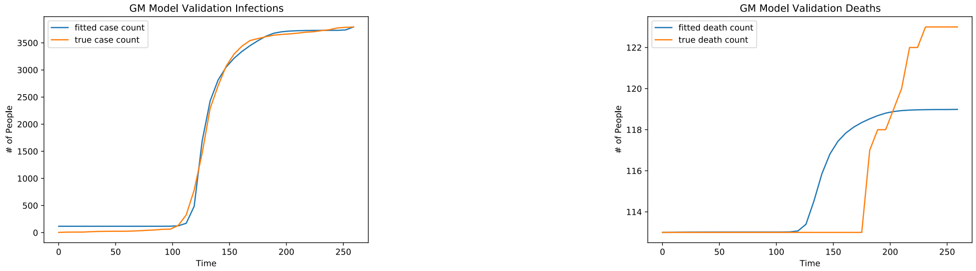

(a) Our model fit to Gambia's infection counts (b) Our model fit to Gambia's death counts

Figure S4: Gambia COVID-19 Dynamics Fitting

with low initial infections, possibly due to undercounting, we see that our model shows delayed infection and death curves in Figure S5.

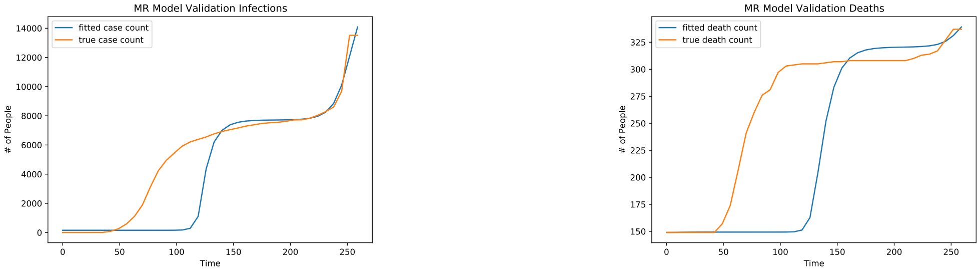

(a) Our model fit to Mauritania's infection counts (b) Our model fit to Mauritania's death counts

Figure S5: Mauritania COVID-19 Dynamics Fitting

4.2 Enumeration of parameters used for numerical evaluations

The sizes of different groups are determined as specified in Section 4.1. Contact rates between these groups were determined in one of two ways: (1) via modified survey-based contact matrices from [26] or (2) by regressing our model to fit real world infection and death counts from [21]. The latter case is described in Section 4.1. Here we discuss the former.

Country-specific contact matrices are obtained from [26]. These are provided in age increments of five years. To produce our contact matrices, we take the contact rate of the high risk group to be the weighted average of the contact rates of each 65+ age group with weight corresponding to the population of each age group obtained from [21]. The contact rate of the high contact group is set to be a multiple of that of the average contact rate over working age individuals. The contact multiple for the high contact group is varied between 1.2 and 6. Finally, R_0 is computed via the next generation matrix method and normalized (by scalar multiplication of the contact matrix) to a fixed value in our parameter range (following the methodology of [27]). By default we set the vaccination capacity constraint to be at 0.5% of the total population daily unless otherwise specified [28]. We obtain the demographic data from census data as in the previous section. We consider the level of initial infection specified as the fraction of the overall populace. Initial infections are varied between 0.1% and 1% – this range captures the seroprevalence upon the introduction of vaccines in all US States and nearly all countries for which data was available[2] [21]. The default assumption is that initial infections are seeded only in the baseline group; we explicitly specify when we deviate from the default assumptions. We fix the efficacy of vaccine at preventing infection, symptoms, hospitalization, and death as 50%, 70%, 80%, and 90%, respectively unless otherwise specified [12]. We used the contact rates obtained from the real evolution of the pandemic as in the previous section. We also use additional contact rates from ranges of contact rates within and across age groups in 139 countries obtained from surveys and the sizes of the different groups [26]. Over all, the large range of contact rates we consider capture varying degree of implementation and compliance with non-pharmaceutical interventions (NPIs) such as social distancing and lockdowns in different US states and the 139 countries. Finally we vary the vaccine efficacy, that is, the rates at which vaccinated individuals become infected, symptomatic, hospitalized, and deceased, over ranges drawn from clinical studies of various vaccines [12, 13]. Over all these parameter ranges, our model was instantiated and run on a fine grid of approximately $911,250$ settings. The associated ranges are included below and are used for the results detailed throughout Sections 4.2, 4.3, 4.7, and 4.8.

Table 4: Model Instances

Parameter	Setting(s)	Ref.
Contact matrices	150 country estimates	[26]
COVID-19 Variants	{alpha, delta, omicron}	[30], [31]
R_0	{1, 1.5, 2, 2.5, 3}	[32]
NPI Efficacy	{0, 0.3, 0.6}	[33]
Initial Infections	[0.1%, 0.25%, 0.5%, 0.75% 1%]	[21]
Vaccine Efficacy	baseline, baseline ± 20%	[12],[13]
High Contact Population Size	{10%, 15%, 20%}	[34]
Transmisibility Multiplier	{1, 1.5, 2}	[35]

For our case studies in Section 4.4, we depart slightly from the above methodology where necessary. In our case study of LMICs, the vaccination capacity constraint is tightened to 0.2% to reflect scarcity [28]. For the prison and nursing home case studies, population demography was obtained from the Bureau of Prisons and the CDC, respectively [36, 37]. Contact matrices for the US Baseline and LMIC were obtained as specified above. For prisons and nursing homes, contact rates were obtained from [38] and [39, 40], respectively.

[2]There is a single country (Gibraltar) which had an initial infection rate higher than our range. In addition, there are countries which are relatively isolated and/or have low population (i.e. New Zealand) that have initial infection rates below our range. Finally, there are a few large countries, notably India and China, which fall below this range, but this may be attributed to suspected undercounting of cases [29].

References

[1] *Coronavirus disease (covid-19): How is it transmitted?* URL: https://www.who.int/news-room/questions-and-answers/item/coronavirus-disease-covid-19-how-is-it-transmitted.

[2] *Covid-19 pandemic planning scenarios.* URL: https://www.cdc.gov/coronavirus/2019-ncov/hcp/planning-scenarios.html.

[3] *Risk for COVID-19 infection, hospitalization, and death by age group.* URL: https://www.cdc.gov/coronavirus/2019-ncov/covid-data/investigations-discovery/hospitalization-death-by-age.html.

[4] *Presymptomatic transmission of SARS-COV-2 - Singapore, January 23–March 16, 2020.* Apr. 2020. URL: https://www.cdc.gov/mmwr/volumes/69/wr/mm6914e1.htm#:~:text=Presymptomatic%5C%20tran.

[5] Jennifer K Bender et al. "Analysis of asymptomatic and presymptomatic transmission in SARS-CoV-2 outbreak, Germany, 2020". In: *Emerging infectious diseases* 27.4 (2021), p. 1159.

[6] *Contact tracing for covid-19.* URL: https://www.cdc.gov/coronavirus/2019-ncov/php/contact-tracing/contact-tracing-plan/contact-tracing.html.

[7] *Interim clinical guidance for management of patients with confirmed coronavirus disease (covid-19).* URL: https://stacks.cdc.gov/view/cdc/88624.

[8] *Mortality analyses.* URL: https://coronavirus.jhu.edu/data/mortality.

[9] *CDC COVID-19 study shows mrna vaccines reduce risk of infection by 91 percent for fully vaccinated people.* June 2021. URL: https://www.cdc.gov/media/releases/2021/p0607-mrna-reduce-risks.html.

[10] National Center for Immunization and Respiratory Diseases. "Science Brief: SARS-CoV-2 Infection-induced and Vaccine-induced Immunity". In: *CDC COVID-19 Science Briefs [Internet]*. Centers for Disease Control and Prevention (US), 2021.

[11] *Comparing the differences between covid-19 vaccines.* URL: https://www.mayoclinic.org/coronavirus-covid-19/vaccine/comparing-vaccines.

[12] Laith J Abu-Raddad, Hiam Chemaitelly, and Adeel A Butt. "Effectiveness of the BNT162b2 Covid-19 Vaccine against the B. 1.1. 7 and B. 1.351 Variants". In: *New England Journal of Medicine* 385.2 (2021), pp. 187–189.

[13] Srinivas Nanduri et al. "Effectiveness of Pfizer-BioNTech and Moderna vaccines in preventing SARS-CoV-2 infection among nursing home residents before and during widespread circulation of the SARS-CoV-2 B. 1.617. 2 (Delta) variant—National Healthcare Safety Network, March 1–August 1, 2021". In: *Morbidity and Mortality Weekly Report* 70.34 (2021), p. 1163.

[14] Victoria Hall et al. "Protection against SARS-CoV-2 after Covid-19 vaccination and previous infection". In: *New England Journal of Medicine* 386.13 (2022), pp. 1207–1220.

[15] Jamie Lopez Bernal et al. "Effectiveness of Covid-19 vaccines against the B. 1.617. 2 (Delta) variant". In: *New England Journal of Medicine* 385.7 (2021), pp. 585–594.

[16] Nicola Mulberryid et al. "Vaccine rollout strategies: The case for vaccinating essential workers early". In: *PLOS Global Public Health* 1 (10 Oct. 2021), e0000020. ISSN: 2767-3375. DOI: 10.1371/JOURNAL.PGPH.0000020. URL: https://journals.plos.org/globalpublichealth/article?id=10.1371/journal.pgph.0000020.

[17] Diego S Silva and Maxwell J Smith. "Social distancing, social justice, and risk during the COVID-19 pandemic". In: *Canadian journal of public health* 111 (2020), pp. 459–461.

[18] Lisa R Fortuna et al. "Inequity and the disproportionate impact of COVID-19 on communities of color in the United States: The need for a trauma-informed social justice response." In: *Psychological Trauma: Theory, Research, Practice, and Policy* 12.5 (2020), p. 443.

[19] Lev Semenovich Pontryagin. *Mathematical theory of optimal processes.* CRC press, 1987.

[20] Dieter Grass et al. *Optimal control of nonlinear processes with applications in drugs,corruption, and terror.* Springer, 2010.

[21] O. Wahltinez et al. "COVID-19 Open-Data: curating a fine-grained, global-scale data repository for SARS-CoV-2". In: (2020). Work in progress. URL: https://goo.gle/covid-19-open-data.

[22] *Report COVID-19: Essential Workers in the States.* URL: https://www.ncsl.org/labor-and-employment/covid-19-essential-workers-in-the-states.

[23] *US states with the most essential workers.* Dec. 2021. URL: https://unitedwaynca.org/blog/us-states-with-the-most-ssential-workers/.

[24] URL: https://bbs.portal.gov.bd/sites/default/files/files/bbs.portal.gov.bd/page/057b0f3b_a9e8_4fde_b3a6_6daec3853586/2021-12-02-10-01-a5b3adcd2ea20db89d4bae0c90bd86cf.pdf.

[25] *Population, total.* URL: https://data.worldbank.org/indicator/SP.POP.TOTL.

[26] Kiesha Prem, Alex R Cook, and Mark Jit. "Projecting social contact matrices in 152 countries using contact surveys and demographic data". In: *PLoS computational biology* 13.9 (2017), e1005697.

[27] Kate M Bubar et al. "Model-informed COVID-19 vaccine prioritization strategies by age and serostatus". In: *Science* 371.6532 (2021), pp. 916–921.

[28] Edouard Mathieu et al. "A global database of COVID-19 vaccinations". In: *Nature human behaviour* 5.7 (2021), pp. 947–953.

[29] Claire Klobucista. *By how much are countries underreporting COVID-19 cases and deaths?* URL: https://www.cfr.org/in-brief/how-much-are-countries-underreporting-covid-19-cases-and-deaths.

[30] Yusha Araf et al. "Omicron variant of SARS-CoV-2: genomics, transmissibility, and responses to current COVID-19 vaccines". In: *Journal of medical virology* 94.5 (2022), pp. 1825–1832.

[31] Kathy Katella. *Omicron, Delta, Alpha, and more: What to know about the coronavirusnbsp;variants.* Feb. 2023. URL: https://www.yalemedicine.org/news/covid-19-variants-of-concern-omicron.

[32] Joe Hilton and Matt J Keeling. "Estimation of country-level basic reproductive ratios for novel Coronavirus (SARS-CoV-2/COVID-19) using synthetic contact matrices". In: *PLoS computational biology* 16.7 (2020), e1008031.

[33] Nadya Johanna, Henrico Citrawijaya, and Grace Wangge. "Mass screening vs lockdown vs combination of both to control COVID-19: A systematic review". In: *Journal of public health research* 9.4 (2020), jphr–2020.

[34] Celine McNicholas and Margaret Poydock. *Who are essential workers?: A comprehensive look at their wages, demographics, and unionization rates.* May 2020. URL: https://www.epi.org/blog/who-are-essential-workers-a-comprehensive-look-at-their-wages-demographics-and-unionization-rates/.

[35] J O'grady et al. *Tuberculosis in prisons: anatomy of global neglect.* 2011.

[36] *Federal Bureau of Prisons.* URL: https://www.bop.gov/about/statistics/population_statistics.jsp.

[37] *FASTSTATS - Residential Care Community.* Dec. 2022. URL: https://www.cdc.gov/nchs/fastats/residential-care-communities.htm.

[38] Martial L Ndeffo-Mbah et al. "Dynamic models of infectious disease transmission in prisons and the general population". In: *Epidemiologic reviews* 40.1 (2018), pp. 40–57.

[39] Andrew T Levin et al. "COVID-19 prevalence and mortality in longer-term care facilities". In: *European Journal of Epidemiology* (2022), pp. 1–8.

[40] Courtney H Van Houtven, Nathan A Boucher, and Walter D Dawson. “Impact of the COVID-19 outbreak on long-term care in the United States”. In: *International Long-Term Care Policy Network* (2020).

[41] Jack H Buckner, Gerardo Chowell, and Michael R Springborn. “Dynamic prioritization of COVID-19 vaccines when social distancing is limited for essential workers”. In: *Proceedings of the National Academy of Sciences* 118.16 (2021).

[42] Rajan Patel, Ira M Longini Jr, and M Elizabeth Halloran. “Finding optimal vaccination strategies for pandemic influenza using genetic algorithms”. In: *Journal of theoretical biology* 234.2 (2005), pp. 201–212.

[43] URL: https://www.cdc.gov/covid/hcp/clinical-care/underlying-conditions.html#cdc_generic_section_6-key-findings-from-one-large-cross-sectional-study.

[44] Oct. 2022. URL: https://www.cdc.gov/nchs/products/databriefs/db446.htm.

[45] Jan. 2024. URL: https://www.ssa.gov/pubs/EN-05-10043.pdf.

[46] URL: https://www.census.gov/popclock/.

[47] Dana Braga and Richard Fry. *1. the growth of the older workforce*. Dec. 2023. URL: https://www.pewresearch.org/social-trends/2023/12/14/the-growth-of-the-older-workforce/#:~:text=Some%2019%25%20of%20adults%20ages,18%25%20of%20older%20Americans%20worked..

[48] Jr. John J. DiIulio et al. *Public service and the Federal Government*. June 2023. URL: https://www.brookings.edu/articles/public-service-and-the-federal-government/#:~:text=and%20small%20businesses.-,Across%20the%20U.S.%2C%20nearly%2024%20million%20people%E2%80%94a%20little%20over,in%20state%20and%20local%20governments..

[49] URL: https://www.bls.gov/careeroutlook/2017/article/older-workers.htm.

[50] Samuel Stebbins, Grant Suneson, and Douglas A. McIntyre. *These are the jobs with the oldest workforces in the United States, from farmers to shuttle drivers*. Oct. 2021. URL: https://www.usatoday.com/story/news/nation/2021/10/26/these-jobs-have-oldest-workforce-country/6166671001/.

[51] URL: https://www.lung.org/lung-health-diseases/lung-disease-lookup/asthma/learn-about-asthma/types/severe-asthma#:~:text=Diagnosing%20Severe%20Asthma&text=Of%20the%20more%20than%2025,or%20are%20just%20uncontrolled%20asthma..

[52] Eileen Wang et al. “Characterization of severe asthma worldwide: data from the International Severe Asthma Registry”. In: *Chest* 157.4 (2020), pp. 790–804.

[53] URL: https://www.cancer.org/cancer/managing-cancer/side-effects/infections/preventing-infections-in-people-with-cancer.html.

[54] Eric S Donkor. “Stroke in the 21st century: a snapshot of the burden, epidemiology, and quality of life”. In: *Stroke research and treatment* 2018.1 (2018), p. 3238165.

[55] Mohammed Yousufuddin and Nathan Young. “Aging and ischemic stroke”. In: *Aging (Albany NY)* 11.9 (2019), p. 2542.

[56] URL: https://esrdnetworks.org/resources-news/national-esrd-census-data/#:~:text=National%20ESRD%20Data%20as%20of,%5Badd%20access%20date%20here%5D..

[57] Centers for Disease Control, Prevention, et al. “Chronic kidney disease in the United States, 2023”. In: *Atlanta, GA: US Department of Health and Human Services, Centers for Disease Control and Prevention* (2023).

[58] URL: https://www.lung.org/about-us/our-impact#:~:text=More%20than%2035%20million%20people,living%20with%20a%20lung%20disease..

[59] Oct. 2024. URL: https://aafa.org/asthma/asthma-facts/#:~:text=Asthma%20can%20be%20deadly%20if,of%20Asthma%20Attacks%20in%20Children?.

[60] Hope Gillette. *Working with COPD: What are the options?* Jan. 2023. URL: https://www.healthline.com/health/can-you-work-with-copd.

[61] Nov. 2023. URL: https://www.cdc.gov/nchs/fastats/liver-disease.htm#:~:text=Number%20of%20adults%20age%2018,with%20diagnosed%20liver%20disease:%201.8%25.

[62] Daniela P Ladner et al. "Increasing prevalence of cirrhosis among insured adults in the United States, 2012–2018". In: *PloS one* 19.2 (2024), e0298887.

[63] URL: https://www.cff.org/intro-cf/about-cystic-fibrosis#:~:text=the%20United%20States:-,There%20are%20close%20to%2040%2C000%20children%20and%20adults%20living%20with,is%20age%2018%20or%20older..

[64] Anjali D Deshpande, Marcie Harris-Hayes, and Mario Schootman. "Epidemiology of diabetes and diabetes-related complications". In: *Physical therapy* 88.11 (2008), pp. 1254–1264.

[65] Taylor M Shockey, Rebecca J Tsai, and Pyone Cho. "Prevalence of diagnosed diabetes among employed us adults by demographic characteristics and occupation, 36 states, 2014 to 2018". In: *Journal of occupational and environmental medicine* 63.4 (2021), pp. 302–310.

[66] Anna Milanesi and Jane E Weinreb. "Diabetes in the elderly". In: (2015).

[67] Biykem Bozkurt et al. "Heart failure epidemiology and outcomes statistics: a report of the Heart Failure Society of America". In: (2023).

[68] Michael W Rich. "Heart failure in the 21st century: a cardiogeriatric syndrome". In: *The Journals of Gerontology Series A: Biological Sciences and Medical Sciences* 56.2 (2001), pp. M88–M96.

[69] URL: https://www.hiv.gov/hiv-basics/overview/data-and-trends/statistics#:~:text=At%20year%2Dend%202022%2C%20an,to%20the%20latest%20CDC%20data:.

[70] URL: https://www.hiv.gov/hiv-basics/living-well-with-hiv/taking-care-of-yourself/aging-with-hiv#:.

[71] Samuel D Emmerich et al. "Obesity and Severe Obesity Prevalence in Adults: United States, August 2021–August 2023". In: (2024).

[72] Yizhe Lim and Joshua Boster. "Obesity and comorbid conditions". In: (2021).

[73] URL: https://www.niaid.nih.gov/diseases-conditions/primary-immune-deficiency-diseases-pidds.

[74] URL: https://www.cdc.gov/primary-immunodeficiency/about/index.html#:~:text=Washing%20your%20hands%20the%20right,prior%20to%20treatment%20for%20SCID..

[75] Saramoriarty. *U.S. reaches historic milestone of 1 million transplants.* Oct. 2023. URL: https://unos.org/news/u-s-reaches-1-million-transplants/#:~:text=More%20than%20400%2C000%20people%20are%20alive%20today%20with%20a%20functioning%20transplant..

[76] URL: https://www.kidney.org.uk/are-work-and-a-normal-life-possible-after-a-transplant#:~:text=It%20is%20usual%20to%20take,directly%20on%20to%20the%20kidney..

[77] Paula M Williams. "Tuberculosis—United States, 2023". In: *MMWR. Morbidity and Mortality Weekly Report* 73 (2024).

[78] Aug. 2024. URL: https://www.dhs.wisconsin.gov/tb/precautions.htm#:~:text=Patients%20with%20confirmed%20infectious%20TB,is%20deemed%20to%20be%20noninfectious..

[79] Oct. 2024. URL: https://www.kff.org/other/state-indicator/distribution-by-age/?currentTimeframe=0&sortModel=%7B%22colId%22%3A%22Location%22%2C%22sort%22%3A%22asc%22%7D.
