# Supplementary material for: Protect or prevent? A practicable framework for the dilemmas of COVID-19 vaccine prioritization
Source: PLoS One. 2025 Jan 22;20(1):e0316294. doi: 10.1371/journal.pone.0316294 (PMC11753641; doi:10.1371/journal.pone.0316294)
Supplement: S5 Appendix — (PDF) [file pone.0316294.s005.pdf]

Protect or prevent? A practicable framework for the dilemmas of COVID-19 vaccine prioritization
Supporting Information

Raghu Arghal[1*], Harvey Rubin[2], Shirin Saeedi Bidokhti[1], Saswati Sarkar[1]

March 2023

1 Department of Electrical and Systems Engineering, University of Pennsylvania, Philadelphia, PA, United States

2 Division of Infectious Diseases, Department of Medicine, University of Pennsylvania School of Medicine, Philadelphia, PA, United States

***** Corresponding Author (rarghal@seas.upenn@edu)

5 Runtime performance and comparison

Throughout our paper we refer to the computational efficiency of our framework (see Sections 2, 4.2, 5). Here we evaluate the runtime of computing the optimal vaccination policy over the landscape of instances defined in Section 4.2. As shown in Figure S6, the runtimes of our algorithm, omitting the small number of instance that reached the cutoff time of 500 seconds, were heavily concentrated under 10 seconds. This is a drastic improvement over simulation based techniques or more complex optimization protocols and allows us to survey broad parameter landscapes to map optimal policies as presented in Section 4.3.

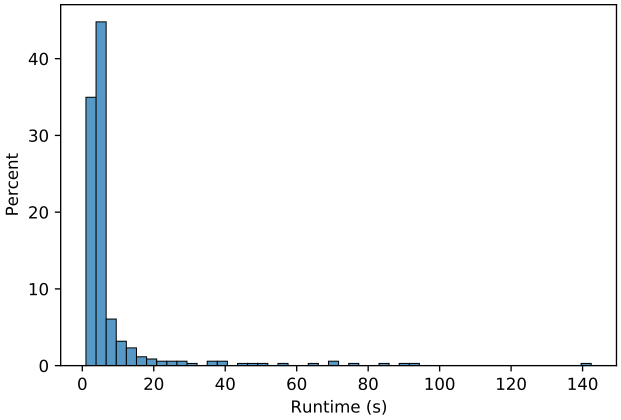

Figure S6: Histogram of runtimes for our optimal control-based vaccine prioritization method

To our knowledge, the closest work to consider the optimal selection of dynamic COVID-19 vaccination policies is the paper authored by Buckner et. al. [41]. Here they utilize a genetic algorithm based on the work in [42] followed by a simulated annealing algorithm to more precisely identify their optimal. Genetic algorithms and simulated annealing can optimize only discrete variables but not arbitrary functions of time. Thus these can optimize vaccination strategies only among a reduced strategy space, namely, among strategies that allocate piece-wise constant vaccination rates to different groups. Specifically, the overall time horizon under consideration is partitioned into intervals and the fraction of overall capacity allocated to different groups in each interval are considered as constants over time in the interval. The fractions in different intervals are the optimization variables, which are chosen optimally, to optimize the public health objective in this restricted policy space. The policy space becomes closer to the space of all vaccination strategies as the number of intervals increase. But the computation time for the genetic algorithm is exponential in the number of time intervals [42]. Thus the computation time increases rapidly as the number of intervals increase. Buckner et. al. considered a time horizon of 6 months and the interval size of 1 month; thus the vaccination rates across the groups they consider can change only once a month [41]. But even with such coarse granularity their genetic algorithm step alone can take hours to converge to an optimal in a single instance, even without including the subsequent simulated annealing. Since the computation time is exponential in the number of time intervals, runtime becomes intractable when considering highly dynamic policies as shown in Table 5, which rules out obtaining solutions using current computing capabilities and thereby using such solutions even as benchmarks for comparison let alone for actual deployment. This is an inherent limitation on the ability of genetic algorithms to consider highly dynamic policies. In contrast, our computation framework optimizes the vaccination policy in much broader policy space than the above, among policies whose choices can arbitrarily vary at any time granularity; still our framework yields the optimal policy in seconds. Even considering policies that can change allocations only once every month, Buckner et. al. presented the optimal in this reduced space only for a small number of instances [41],

Runtime Comparison		
Length of Decision Intervals (days)	Our Method Mean Runtime (s)	Buckner et al. Mean Runtime (s)
90	2.86	2470.71
30	9.59	3076.86
10	10.50	6277.54 (projected)
7	14.54	8258.43 (projected)
1	160.59	4.30×10^7 (projected)

Table 5: This table shows the mean runtime of determining the optimal vaccination policy when instantiated on different contact matrices and demography [21, 26]. For shorter decision interval lengths, the runtime of Buckner et al.'s algorithm is projected using an exponential fit with the number of decision variables as the independent variable because their algorithm is exponential in this dimension.

perhaps due to the long computation time of their method, whereas we could present results for large landscapes of 911,250 instances involving variations of parameters in large realistic ranges (Section 4). Large landscapes allow for more reliable conclusions on how the optimal strategy and the optimal value of the public health objective changes with variations in parameter values; large landscapes also help identify near-optimal easy-to-deploy vaccination strategies which we accomplish. Next, generalizations to consider several attributes of practicality inevitably increases the state space of the set of trajectory (e.g. when breakthrough infections and reinfections are considered), and in many instances the number of functions that need to be optimized (e.g. for two dose vaccination). All these increase the computation time for each instance further and therefore renders the computations even more formidable if they are involved in the simplest case. Thus computational tractability of the basic framework is imperative for its adaptability to more involved albeit realistic settings. Next, most works in this genre (including Buckner *et al* partition the populace into a larger number of groups than us, and based on a different criteria (age and in one case profession) [41, 27]. Our computation time for each instance is lower also because we consider fewer groups (though the primary reason is the choice of the optimal control formulation rather than the genetic algorithm and simulated annealing combo). As mentioned before, our model is able to accurately predict the evolution and spread of COVID-19 in geographical units of different scale and locations all over the world, despite consideration of partition criteria that yields fewer groups. Finally, our optimal strategy is structurally different from that obtained by Buckner *et al* because of 1) the significant difference in time granularity over which vaccination rates allocated to different groups can change (ours can change any time, theirs can change only once a month) and 2) different criteria for partition of the populace into groups. For example, the policy obtained by Buckner et al is always mixed, i.e., vaccinates multiple groups simultaneously, whereas we analytically argue that the death count does not increase if the policy space is limited to those that vaccinate one group at a time, while the susceptibles in each group exceeds the vaccine capacity (i.e. while each group has enough individuals to vaccinate if it is given the full vaccination capacity). If we optimize in a restricted policy space where the allocations can change only once a month, our optimal strategy is also mixed from the start.

References

[1] *Coronavirus disease (covid-19): How is it transmitted?* URL: https://www.who.int/news-room/questions-and-answers/item/coronavirus-disease-covid-19-how-is-it-transmitted.

[2] *Covid-19 pandemic planning scenarios.* URL: https://www.cdc.gov/coronavirus/2019-ncov/hcp/planning-scenarios.html.

[3] *Risk for COVID-19 infection, hospitalization, and death by age group.* URL: https://www.cdc.gov/coronavirus/2019-ncov/covid-data/investigations-discovery/hospitalization-death-by-age.html.

[4] *Presymptomatic transmission of SARS-COV-2 - Singapore, January 23–March 16, 2020.* Apr. 2020. URL: https://www.cdc.gov/mmwr/volumes/69/wr/mm6914e1.htm#:~:text=Presymptomatic%5C%20tran.

[5] Jennifer K Bender et al. "Analysis of asymptomatic and presymptomatic transmission in SARS-CoV-2 outbreak, Germany, 2020". In: *Emerging infectious diseases* 27.4 (2021), p. 1159.

[6] *Contact tracing for covid-19.* URL: https://www.cdc.gov/coronavirus/2019-ncov/php/contact-tracing/contact-tracing-plan/contact-tracing.html.

[7] *Interim clinical guidance for management of patients with confirmed coronavirus disease (covid-19).* URL: https://stacks.cdc.gov/view/cdc/88624.

[8] *Mortality analyses.* URL: https://coronavirus.jhu.edu/data/mortality.

[9] *CDC COVID-19 study shows mrna vaccines reduce risk of infection by 91 percent for fully vaccinated people.* June 2021. URL: https://www.cdc.gov/media/releases/2021/p0607-mrna-reduce-risks.html.

[10] National Center for Immunization and Respiratory Diseases. "Science Brief: SARS-CoV-2 Infection-induced and Vaccine-induced Immunity". In: *CDC COVID-19 Science Briefs [Internet]*. Centers for Disease Control and Prevention (US), 2021.

[11] *Comparing the differences between covid-19 vaccines.* URL: https://www.mayoclinic.org/coronavirus-covid-19/vaccine/comparing-vaccines.

[12] Laith J Abu-Raddad, Hiam Chemaitelly, and Adeel A Butt. "Effectiveness of the BNT162b2 Covid-19 Vaccine against the B. 1.1. 7 and B. 1.351 Variants". In: *New England Journal of Medicine* 385.2 (2021), pp. 187–189.

[13] Srinivas Nanduri et al. "Effectiveness of Pfizer-BioNTech and Moderna vaccines in preventing SARS-CoV-2 infection among nursing home residents before and during widespread circulation of the SARS-CoV-2 B. 1.617. 2 (Delta) variant—National Healthcare Safety Network, March 1–August 1, 2021". In: *Morbidity and Mortality Weekly Report* 70.34 (2021), p. 1163.

[14] Victoria Hall et al. "Protection against SARS-CoV-2 after Covid-19 vaccination and previous infection". In: *New England Journal of Medicine* 386.13 (2022), pp. 1207–1220.

[15] Jamie Lopez Bernal et al. "Effectiveness of Covid-19 vaccines against the B. 1.617. 2 (Delta) variant". In: *New England Journal of Medicine* 385.7 (2021), pp. 585–594.

[16] Nicola Mulberryid et al. "Vaccine rollout strategies: The case for vaccinating essential workers early". In: *PLOS Global Public Health* 1 (10 Oct. 2021), e0000020. ISSN: 2767-3375. DOI: 10.1371/JOURNAL.PGPH.0000020. URL: https://journals.plos.org/globalpublichealth/article?id=10.1371/journal.pgph.0000020.

[17] Diego S Silva and Maxwell J Smith. "Social distancing, social justice, and risk during the COVID-19 pandemic". In: *Canadian journal of public health* 111 (2020), pp. 459–461.

[18] Lisa R Fortuna et al. "Inequity and the disproportionate impact of COVID-19 on communities of color in the United States: The need for a trauma-informed social justice response." In: *Psychological Trauma: Theory, Research, Practice, and Policy* 12.5 (2020), p. 443.

[19] Lev Semenovich Pontryagin. *Mathematical theory of optimal processes*. CRC press, 1987.

[20] Dieter Grass et al. *Optimal control of nonlinear processes with applications in drugs,corruption, and terror*. Springer, 2010.

[21] O. Wahltinez et al. "COVID-19 Open-Data: curating a fine-grained, global-scale data repository for SARS-CoV-2". In: (2020). Work in progress. URL: https://goo.gle/covid-19-open-data.

[22] *Report COVID-19: Essential Workers in the States*. URL: https://www.ncsl.org/labor-and-employment/covid-19-essential-workers-in-the-states.

[23] *US states with the most essential workers*. Dec. 2021. URL: https://unitedwaynca.org/blog/us-states-with-the-most-ssential-workers/.

[24] URL: https://bbs.portal.gov.bd/sites/default/files/files/bbs.portal.gov.bd/page/057b0f3b_a9e8_4fde_b3a6_6daec3853586/2021-12-02-10-01-a5b3adcd2ea20db89d4bae0c90bd86cf.pdf.

[25] *Population, total*. URL: https://data.worldbank.org/indicator/SP.POP.TOTL.

[26] Kiesha Prem, Alex R Cook, and Mark Jit. "Projecting social contact matrices in 152 countries using contact surveys and demographic data". In: *PLoS computational biology* 13.9 (2017), e1005697.

[27] Kate M Bubar et al. "Model-informed COVID-19 vaccine prioritization strategies by age and serostatus". In: *Science* 371.6532 (2021), pp. 916–921.

[28] Edouard Mathieu et al. "A global database of COVID-19 vaccinations". In: *Nature human behaviour* 5.7 (2021), pp. 947–953.

[29] Claire Klobucista. *By how much are countries underreporting COVID-19 cases and deaths?* URL: https://www.cfr.org/in-brief/how-much-are-countries-underreporting-covid-19-cases-and-deaths.

[30] Yusha Araf et al. "Omicron variant of SARS-CoV-2: genomics, transmissibility, and responses to current COVID-19 vaccines". In: *Journal of medical virology* 94.5 (2022), pp. 1825–1832.

[31] Kathy Katella. *Omicron, Delta, Alpha, and more: What to know about the coronavirusnbsp;variants*. Feb. 2023. URL: https://www.yalemedicine.org/news/covid-19-variants-of-concern-omicron.

[32] Joe Hilton and Matt J Keeling. "Estimation of country-level basic reproductive ratios for novel Coronavirus (SARS-CoV-2/COVID-19) using synthetic contact matrices". In: *PLoS computational biology* 16.7 (2020), e1008031.

[33] Nadya Johanna, Henrico Citrawijaya, and Grace Wangge. "Mass screening vs lockdown vs combination of both to control COVID-19: A systematic review". In: *Journal of public health research* 9.4 (2020), jphr–2020.

[34] Celine McNicholas and Margaret Poydock. *Who are essential workers?: A comprehensive look at their wages, demographics, and unionization rates*. May 2020. URL: https://www.epi.org/blog/who-are-essential-workers-a-comprehensive-look-at-their-wages-demographics-and-unionization-rates/.

[35] J O'grady et al. *Tuberculosis in prisons: anatomy of global neglect*. 2011.

[36] *Federal Bureau of Prisons*. URL: https://www.bop.gov/about/statistics/population_statistics.jsp.

[37] *FASTSTATS - Residential Care Community*. Dec. 2022. URL: https://www.cdc.gov/nchs/fastats/residential-care-communities.htm.

[38] Martial L Ndeffo-Mbah et al. "Dynamic models of infectious disease transmission in prisons and the general population". In: *Epidemiologic reviews* 40.1 (2018), pp. 40–57.

[39] Andrew T Levin et al. "COVID-19 prevalence and mortality in longer-term care facilities". In: *European Journal of Epidemiology* (2022), pp. 1–8.

[40] Courtney H Van Houtven, Nathan A Boucher, and Walter D Dawson. "Impact of the COVID-19 outbreak on long-term care in the United States". In: *International Long-Term Care Policy Network* (2020).

[41] Jack H Buckner, Gerardo Chowell, and Michael R Springborn. "Dynamic prioritization of COVID-19 vaccines when social distancing is limited for essential workers". In: *Proceedings of the National Academy of Sciences* 118.16 (2021).

[42] Rajan Patel, Ira M Longini Jr, and M Elizabeth Halloran. "Finding optimal vaccination strategies for pandemic influenza using genetic algorithms". In: *Journal of theoretical biology* 234.2 (2005), pp. 201–212.

[43] URL: https://www.cdc.gov/covid/hcp/clinical-care/underlying-conditions.html#cdc_generic_section_6-key-findings-from-one-large-cross-sectional-study.

[44] Oct. 2022. URL: https://www.cdc.gov/nchs/products/databriefs/db446.htm.

[45] Jan. 2024. URL: https://www.ssa.gov/pubs/EN-05-10043.pdf.

[46] URL: https://www.census.gov/popclock/.

[47] Dana Braga and Richard Fry. *1. the growth of the older workforce*. Dec. 2023. URL: https://www.pewresearch.org/social-trends/2023/12/14/the-growth-of-the-older-workforce/#:~:text=Some%2019%25%20of%20adults%20ages,18%25%20of%20older%20Americans%20worked..

[48] Jr. John J. DiIulio et al. *Public service and the Federal Government*. June 2023. URL: https://www.brookings.edu/articles/public-service-and-the-federal-government/#:~:text=and%20small%20businesses.-,Across%20the%20U.S.%2C%20nearly%2024%20million%20people%E2%80%94a%20little%20over,in%20state%20and%20local%20governments..

[49] URL: https://www.bls.gov/careeroutlook/2017/article/older-workers.htm.

[50] Samuel Stebbins, Grant Suneson, and Douglas A. McIntyre. *These are the jobs with the oldest workforces in the United States, from farmers to shuttle drivers*. Oct. 2021. URL: https://www.usatoday.com/story/news/nation/2021/10/26/these-jobs-have-oldest-workforce-country/6166671001/.

[51] URL: https://www.lung.org/lung-health-diseases/lung-disease-lookup/asthma/learn-about-asthma/types/severe-asthma#:~:text=Diagnosing%20Severe%20Asthma&text=Of%20the%20more%20than%2025,or%20are%20just%20uncontrolled%20asthma..

[52] Eileen Wang et al. "Characterization of severe asthma worldwide: data from the International Severe Asthma Registry". In: *Chest* 157.4 (2020), pp. 790–804.

[53] URL: https://www.cancer.org/cancer/managing-cancer/side-effects/infections/preventing-infections-in-people-with-cancer.html.

[54] Eric S Donkor. "Stroke in the 21st century: a snapshot of the burden, epidemiology, and quality of life". In: *Stroke research and treatment* 2018.1 (2018), p. 3238165.

[55] Mohammed Yousufuddin and Nathan Young. "Aging and ischemic stroke". In: *Aging (Albany NY)* 11.9 (2019), p. 2542.

[56] URL: https://esrdnetworks.org/resources-news/national-esrd-census-data/#:~:text=National%20ESRD%20Data%20as%20of,%5Badd%20access%20date%20here%5D..

[57] Centers for Disease Control, Prevention, et al. "Chronic kidney disease in the United States, 2023". In: *Atlanta, GA: US Department of Health and Human Services, Centers for Disease Control and Prevention* (2023).

[58] URL: https://www.lung.org/about-us/our-impact#:~:text=More%20than%2035%20million%20people,living%20with%20a%20lung%20disease..

[59] Oct. 2024. URL: https://aafa.org/asthma/asthma-facts/#:~:text=Asthma%20can%20be%20deadly%20if,of%20Asthma%20Attacks%20in%20Children?.

[60] Hope Gillette. *Working with COPD: What are the options?* Jan. 2023. URL: https://www.healthline.com/health/can-you-work-with-copd.

[61] Nov. 2023. URL: https://www.cdc.gov/nchs/fastats/liver-disease.htm#:~:text=Number%20of%20adults%20age%2018,with%20diagnosed%20liver%20disease:%201.8%25.

[62] Daniela P Ladner et al. "Increasing prevalence of cirrhosis among insured adults in the United States, 2012–2018". In: *PloS one* 19.2 (2024), e0298887.

[63] URL: https://www.cff.org/intro-cf/about-cystic-fibrosis#:~:text=the%20United%20States:-,There%20are%20close%20to%2040%2C000%20children%20and%20adults%20living%20with,is%20age%2018%20or%20older..

[64] Anjali D Deshpande, Marcie Harris-Hayes, and Mario Schootman. "Epidemiology of diabetes and diabetes-related complications". In: *Physical therapy* 88.11 (2008), pp. 1254–1264.

[65] Taylor M Shockey, Rebecca J Tsai, and Pyone Cho. "Prevalence of diagnosed diabetes among employed us adults by demographic characteristics and occupation, 36 states, 2014 to 2018". In: *Journal of occupational and environmental medicine* 63.4 (2021), pp. 302–310.

[66] Anna Milanesi and Jane E Weinreb. "Diabetes in the elderly". In: (2015).

[67] Biykem Bozkurt et al. "Heart failure epidemiology and outcomes statistics: a report of the Heart Failure Society of America". In: (2023).

[68] Michael W Rich. "Heart failure in the 21st century: a cardiogeriatric syndrome". In: *The Journals of Gerontology Series A: Biological Sciences and Medical Sciences* 56.2 (2001), pp. M88–M96.

[69] URL: https://www.hiv.gov/hiv-basics/overview/data-and-trends/statistics#:~:text=At%20year%2Dend%202022%2C%20an,to%20the%20latest%20CDC%20data:.

[70] URL: https://www.hiv.gov/hiv-basics/living-well-with-hiv/taking-care-of-yourself/aging-with-hiv#:.

[71] Samuel D Emmerich et al. "Obesity and Severe Obesity Prevalence in Adults: United States, August 2021–August 2023". In: (2024).

[72] Yizhe Lim and Joshua Boster. "Obesity and comorbid conditions". In: (2021).

[73] URL: https://www.niaid.nih.gov/diseases-conditions/primary-immune-deficiency-diseases-pidds.

[74] URL: https://www.cdc.gov/primary-immunodeficiency/about/index.html#:~:text=Washing%20your%20hands%20the%20right,prior%20to%20treatment%20for%20SCID..

[75] Saramoriarty. *U.S. reaches historic milestone of 1 million transplants*. Oct. 2023. URL: https://unos.org/news/u-s-reaches-1-million-transplants/#:~:text=More%20than%20400%2C000%20people%20are%20alive%20today%20with%20a%20functioning%20transplant..

[76] URL: https://www.kidney.org.uk/are-work-and-a-normal-life-possible-after-a-transplant#:~:text=It%20is%20usual%20to%20take,directly%20on%20to%20the%20kidney..

[77] Paula M Williams. "Tuberculosis—United States, 2023". In: *MMWR. Morbidity and Mortality Weekly Report* 73 (2024).

[78] Aug. 2024. URL: https://www.dhs.wisconsin.gov/tb/precautions.htm#:~:text=Patients%20with%20confirmed%20infectious%20TB,is%20deemed%20to%20be%20noninfectious..

[79] Oct. 2024. URL: https://www.kff.org/other/state-indicator/distribution-by-age/?currentTimeframe=0&sortModel=%7B%22colId%22%3A%22Location%22%2C%22sort%22%3A%22asc%22%7D.
