# Supplementary material for: Protect or prevent? A practicable framework for the dilemmas of COVID-19 vaccine prioritization
Source: PLoS One. 2025 Jan 22;20(1):e0316294. doi: 10.1371/journal.pone.0316294 (PMC11753641; doi:10.1371/journal.pone.0316294)
Supplement: S6 Appendix — (PDF) [file pone.0316294.s006.pdf]

Protect or prevent? A practicable framework for the dilemmas of COVID-19 vaccine prioritization
Supporting Information

Raghu Arghal[1*], Harvey Rubin[2], Shirin Saeedi Bidokhti[1], Saswati Sarkar[1]

March 2023

1 Department of Electrical and Systems Engineering, University of Pennsylvania, Philadelphia, PA, United States

2 Division of Infectious Diseases, Department of Medicine, University of Pennsylvania School of Medicine, Philadelphia, PA, United States

***** Corresponding Author (rarghal@seas.upenn@edu)

6 Estimation of the size of the overlap group

We seek to estimate the fraction of the US population that is both high contact and high risk. High risk from COVID is induced either by age or some underlying diseases. The concerned underlying diseases have been listed in Table 6 (obtained from [43]). We consider 65 as the age threshold. This is because the CDC has classified persons above this age as high risk for COVID [43] – patients above this age have an over 500% increase in mortality risk upon COVID infection compared to the mortality risk of the low risk population (those below 65 and with no underlying comorbidity) [43]. As such, 81% of US COVID deaths in the first year of the pandemic were individuals over 65 [44]. Besides, 65 is also a common threshold for classification as a senior citizen; for example, it is the minimum age to qualify for Medicare [45].

We resort to some set theoretic terminology to estimate the size the population that is both high contact and high risk. Let A be the set of individuals who are above 65 years of age, B be the set of individuals who have at least one underlying disease in Table 6. Then the high risk group is $A \cup B$ (individuals who belong in either A or B or both). Let C be the set of individuals who are high contact, specifically, who are employed in high contact professions. Thus, the set of individuals who are both high contact and high risk is $(A \cup B) \cap C$. The size of this group is $|(A \cup B) \cap C|$. Then:

$$\begin{aligned} |(A \cup B) \cap C| &= |(A \cap C) \cup (B \cap C)| \\ &= |A \cap C| + |(B \cap C) \setminus (A \cap C)| \\ &= |A \cap C| + |(B \setminus A) \cap C| \end{aligned} \tag{9}$$

Now, $|A \cap C|$ is the set of individuals who are over 65 and are employed in high contact professions. And, $|(B \setminus A) \cap C|$ are the set of individuals who are below 65 but suffer from at least one disease listed in Table 6 and are employed in high contact professions. Note that $B \setminus A$ is the set of individuals who are in B but not in A, and therefore who have at least one underlying disease but whose age is below 65 years of age. Thus, from (9), the size of the population that is both high contact and high risk equals the sum of the sizes of the populations in the following two categories: 1) individuals over 65 who are employed in high contact professions (that is $|A \cap C|$) 2) individuals who are below 65 but suffer from at least one disease listed in Table 6 and are employed in high contact professions (that is, $|(B \setminus A) \cap C|$).

From US census and workforce data we are able to obtain the fraction of the US populace which is above 65 years of age and is working. Economic surveys give us the fraction of US populace working in high contact professions. We could not find the data on the intersection even in the US, however, economic surveys provide us an upper bound on the same. Similarly, from the CDC, we could obtain a list of diseases which increase mortality rate upon contracting COVID (Table 6). From scientific literature, for most of these diseases, we could obtain the age distribution of individuals afflicted with the same (Table 6). We could not however find data on the overlap of the individuals who are employed in high contact professions and suffer from the enumerated diseases for most of those diseases. For those diseases, we therefore assumed that the patients are represented in the same proportion among high contact professions as in the general populace. This grossly overestimates the fraction of individuals who suffer from these diseases and are employed in high contact professions because in many cases the patients will work from home (ie, pursue low contact professions) owing to their lowered immunity, work intermittently, or even not work at all as the disease progresses. The overestimate provides that $|A \cap C|$ is at most 1.02%, and $|(B \setminus A) \cap C|$ is at most 0.87% of the US population, as will be argued in the subsequent paragraphs. Thus, together at most 1.9% of the population in US is both high contact and high risk.

We first estimate the percentage of US population which belongs in the first category (that is $|A \cap C|$). As of 2023 in US the population size is 335 million [46]. There are roughly 11.4 million working people over 65 [47, 48]. Thus, working individuals over the age of 65 make up only about 3.4% of the US population. Further, at least 70% of these working people over 65 are self-employed (16.4%), agricultural workers (11.4%), or have desk jobs (42%) [49] — professions which would not be considered high contact

[50]. This leads to a (possibly significant) overestimate of the percentage of the working population over 65 that is employed in high contact professions, as it assumes that all other professions of working people over 65 are high contact, which is unlikely. Thus, at most 1.02% (0.3×3.4) of the US population is above the age of 65 and employed in high contact professions. That is, $|A \cap C| \leq 1.02\%$.

We now estimate the percentage of the US population that belongs in the second category (that is, $|(B \setminus A) \cap C|$). For several diseases listed in Table 6, the standard health advice is that the afflicted individuals should not pursue high contact professions, we therefore exclude those for obtaining our estimate. For each of the remaining diseases we obtain the percentage of the overall US who suffer from the same and are below 65 years of age, this percentage is the third column entry of the corresponding row. Summing these, we obtain the maximum percentage of the overall populace who suffer from at least one of the listed diseases and are below 65 years of age to be 4.37%. Next, we learn from [34] that the number of high contact workers in US, ie, those who "have a particularly high propensity for spreading COVID because, by nature of their work, they regularly come in contact with large and diverse groups of customers" is 55 million. This is also an upper bound on the number of individuals who are in high contact professions and are below the age of 65. Thus, at most $4.37\% \times \frac{335M}{277M} \times 55M = 2.9M$ million individuals are in the second category where 277 million is the US population aged between 18 and 65 [46]. This is a substantial overestimate because those with the listed serious diseases are less likely than the general populace to have high contact professions. Thus, out of a total population of 335 million, at most 0.87% are in the second category. That is, $|(B \setminus A) \cap C| \leq 0.87\%$.

Condition	US Population %	Age 18 to 65 %	Notes
Severe Asthma	0.8 [51]	0.4 [52]	
Cancer	-	-	Advised to avoid high contact work [53]
Cerebrovascular disease	1.2 [54]	0.3 [55]	
Chronic kidney disease	0.15 [56]	0.01 [57]	
Chronic lung diseases	3.5 [58, 59]	-	"encourage remote, at-home positions" [60]
Chronic liver diseases	1.3 [61]	0.52 [62]	
Cystic fibrosis	0.012 [63]	0.007	
Diabetes with Complications	3.2 [64, 65]	1.9 [66]	
Heart conditions	2 [67]	0.4 [68]	
HIV	0.36 [69]	0.24 [70]	
Morbid Obesity	10 [71]	0.59 [72]	+ See comment in caption
Primary immunodeficiencies	0.15 [73]	-	Advised to avoid high contact work [74]
Transplantation	0.12 [75]	-	Advised to avoid high contact work [76]
Tuberculosis	0.003 [77]	-	Advised to avoid high contact work [78]

Table 6: High Risk Comorbidities: Here we delineate the conditions indicated by the CDC to have higher risk upon COVID infection [43]. The second column is the percent of the total US population who are afflicted with the condition. The third column represents the percentage of the total US population who are afflicted with the condition and between 18 and 65 years of age [46]. Note that we are estimating the population who are below 65, suffer from at least one of the specified conditions, and are employed in a high contact profession. Therefore we do not consider those below 18 because they are not of working age. The content in the third column is obtained by multiplying the second column value with the fraction of the population of that condition between 18 and 65 (obtained from sources indicated in the third column). For cystic fibrosis, we could not find the fraction of patients who are between 18 and 65. Therefore, we apply the general US age population of which 59.2% is between 18 and 65 [79].
+ While the obese, working age population is significant, upwards of 90% of morbidly obese patients have at least one comorbidity among the other conditions in this table (most commonly diabetes, heart conditions, and chronic liver diseases such as non-alcoholic fatty liver disease) [72]. Those 90% are already considered as part of other conditions in this table. We therefore consider the remaining 10% of the working age obese population. Now the corresponding content of the third column is computed as explained above for the other rows.

References

[1] *Coronavirus disease (covid-19): How is it transmitted?* URL: https://www.who.int/news-room/questions-and-answers/item/coronavirus-disease-covid-19-how-is-it-transmitted.

[2] *Covid-19 pandemic planning scenarios.* URL: https://www.cdc.gov/coronavirus/2019-ncov/hcp/planning-scenarios.html.

[3] *Risk for COVID-19 infection, hospitalization, and death by age group.* URL: https://www.cdc.gov/coronavirus/2019-ncov/covid-data/investigations-discovery/hospitalization-death-by-age.html.

[4] *Presymptomatic transmission of SARS-COV-2 - Singapore, January 23–March 16, 2020.* Apr. 2020. URL: https://www.cdc.gov/mmwr/volumes/69/wr/mm6914e1.htm#:~:text=Presymptomatic%5C%20tran.

[5] Jennifer K Bender et al. "Analysis of asymptomatic and presymptomatic transmission in SARS-CoV-2 outbreak, Germany, 2020". In: *Emerging infectious diseases* 27.4 (2021), p. 1159.

[6] *Contact tracing for covid-19.* URL: https://www.cdc.gov/coronavirus/2019-ncov/php/contact-tracing/contact-tracing-plan/contact-tracing.html.

[7] *Interim clinical guidance for management of patients with confirmed coronavirus disease (covid-19).* URL: https://stacks.cdc.gov/view/cdc/88624.

[8] *Mortality analyses.* URL: https://coronavirus.jhu.edu/data/mortality.

[9] *CDC COVID-19 study shows mrna vaccines reduce risk of infection by 91 percent for fully vaccinated people.* June 2021. URL: https://www.cdc.gov/media/releases/2021/p0607-mrna-reduce-risks.html.

[10] National Center for Immunization and Respiratory Diseases. "Science Brief: SARS-CoV-2 Infection-induced and Vaccine-induced Immunity". In: *CDC COVID-19 Science Briefs [Internet]*. Centers for Disease Control and Prevention (US), 2021.

[11] *Comparing the differences between covid-19 vaccines.* URL: https://www.mayoclinic.org/coronavirus-covid-19/vaccine/comparing-vaccines.

[12] Laith J Abu-Raddad, Hiam Chemaitelly, and Adeel A Butt. "Effectiveness of the BNT162b2 Covid-19 Vaccine against the B. 1.1. 7 and B. 1.351 Variants". In: *New England Journal of Medicine* 385.2 (2021), pp. 187–189.

[13] Srinivas Nanduri et al. "Effectiveness of Pfizer-BioNTech and Moderna vaccines in preventing SARS-CoV-2 infection among nursing home residents before and during widespread circulation of the SARS-CoV-2 B. 1.617. 2 (Delta) variant—National Healthcare Safety Network, March 1–August 1, 2021". In: *Morbidity and Mortality Weekly Report* 70.34 (2021), p. 1163.

[14] Victoria Hall et al. "Protection against SARS-CoV-2 after Covid-19 vaccination and previous infection". In: *New England Journal of Medicine* 386.13 (2022), pp. 1207–1220.

[15] Jamie Lopez Bernal et al. "Effectiveness of Covid-19 vaccines against the B. 1.617. 2 (Delta) variant". In: *New England Journal of Medicine* 385.7 (2021), pp. 585–594.

[16] Nicola Mulberryid et al. "Vaccine rollout strategies: The case for vaccinating essential workers early". In: *PLOS Global Public Health* 1 (10 Oct. 2021), e0000020. ISSN: 2767-3375. DOI: 10.1371/JOURNAL.PGPH.0000020. URL: https://journals.plos.org/globalpublichealth/article?id=10.1371/journal.pgph.0000020.

[17] Diego S Silva and Maxwell J Smith. "Social distancing, social justice, and risk during the COVID-19 pandemic". In: *Canadian journal of public health* 111 (2020), pp. 459–461.

[18] Lisa R Fortuna et al. "Inequity and the disproportionate impact of COVID-19 on communities of color in the United States: The need for a trauma-informed social justice response." In: *Psychological Trauma: Theory, Research, Practice, and Policy* 12.5 (2020), p. 443.

[19] Lev Semenovich Pontryagin. *Mathematical theory of optimal processes.* CRC press, 1987.

[20] Dieter Grass et al. *Optimal control of nonlinear processes with applications in drugs,corruption, and terror.* Springer, 2010.

[21] O. Wahltinez et al. "COVID-19 Open-Data: curating a fine-grained, global-scale data repository for SARS-CoV-2". In: (2020). Work in progress. URL: https://goo.gle/covid-19-open-data.

[22] *Report COVID-19: Essential Workers in the States.* URL: https://www.ncsl.org/labor-and-employment/covid-19-essential-workers-in-the-states.

[23] *US states with the most essential workers.* Dec. 2021. URL: https://unitedwaynca.org/blog/us-states-with-the-most-ssential-workers/.

[24] URL: https://bbs.portal.gov.bd/sites/default/files/files/bbs.portal.gov.bd/page/057b0f3b_a9e8_4fde_b3a6_6daec3853586/2021-12-02-10-01-a5b3adcd2ea20db89d4bae0c90bd86cf.pdf.

[25] *Population, total.* URL: https://data.worldbank.org/indicator/SP.POP.TOTL.

[26] Kiesha Prem, Alex R Cook, and Mark Jit. "Projecting social contact matrices in 152 countries using contact surveys and demographic data". In: *PLoS computational biology* 13.9 (2017), e1005697.

[27] Kate M Bubar et al. "Model-informed COVID-19 vaccine prioritization strategies by age and serostatus". In: *Science* 371.6532 (2021), pp. 916–921.

[28] Edouard Mathieu et al. "A global database of COVID-19 vaccinations". In: *Nature human behaviour* 5.7 (2021), pp. 947–953.

[29] Claire Klobucista. *By how much are countries underreporting COVID-19 cases and deaths?* URL: https://www.cfr.org/in-brief/how-much-are-countries-underreporting-covid-19-cases-and-deaths.

[30] Yusha Araf et al. "Omicron variant of SARS-CoV-2: genomics, transmissibility, and responses to current COVID-19 vaccines". In: *Journal of medical virology* 94.5 (2022), pp. 1825–1832.

[31] Kathy Katella. *Omicron, Delta, Alpha, and more: What to know about the coronavirusnbsp;variants.* Feb. 2023. URL: https://www.yalemedicine.org/news/covid-19-variants-of-concern-omicron.

[32] Joe Hilton and Matt J Keeling. "Estimation of country-level basic reproductive ratios for novel Coronavirus (SARS-CoV-2/COVID-19) using synthetic contact matrices". In: *PLoS computational biology* 16.7 (2020), e1008031.

[33] Nadya Johanna, Henrico Citrawijaya, and Grace Wangge. "Mass screening vs lockdown vs combination of both to control COVID-19: A systematic review". In: *Journal of public health research* 9.4 (2020), jphr–2020.

[34] Celine McNicholas and Margaret Poydock. *Who are essential workers?: A comprehensive look at their wages, demographics, and unionization rates.* May 2020. URL: https://www.epi.org/blog/who-are-essential-workers-a-comprehensive-look-at-their-wages-demographics-and-unionization-rates/.

[35] J O'grady et al. *Tuberculosis in prisons: anatomy of global neglect.* 2011.

[36] *Federal Bureau of Prisons.* URL: https://www.bop.gov/about/statistics/population_statistics.jsp.

[37] *FASTSTATS - Residential Care Community.* Dec. 2022. URL: https://www.cdc.gov/nchs/fastats/residential-care-communities.htm.

[38] Martial L Ndeffo-Mbah et al. "Dynamic models of infectious disease transmission in prisons and the general population". In: *Epidemiologic reviews* 40.1 (2018), pp. 40–57.

[39] Andrew T Levin et al. "COVID-19 prevalence and mortality in longer-term care facilities". In: *European Journal of Epidemiology* (2022), pp. 1–8.

[40] Courtney H Van Houtven, Nathan A Boucher, and Walter D Dawson. "Impact of the COVID-19 outbreak on long-term care in the United States". In: *International Long-Term Care Policy Network* (2020).

[41] Jack H Buckner, Gerardo Chowell, and Michael R Springborn. "Dynamic prioritization of COVID-19 vaccines when social distancing is limited for essential workers". In: *Proceedings of the National Academy of Sciences* 118.16 (2021).

[42] Rajan Patel, Ira M Longini Jr, and M Elizabeth Halloran. "Finding optimal vaccination strategies for pandemic influenza using genetic algorithms". In: *Journal of theoretical biology* 234.2 (2005), pp. 201–212.

[43] URL: https://www.cdc.gov/covid/hcp/clinical-care/underlying-conditions.html#cdc_generic_section_6-key-findings-from-one-large-cross-sectional-study.

[44] Oct. 2022. URL: https://www.cdc.gov/nchs/products/databriefs/db446.htm.

[45] Jan. 2024. URL: https://www.ssa.gov/pubs/EN-05-10043.pdf.

[46] URL: https://www.census.gov/popclock/.

[47] Dana Braga and Richard Fry. *1. the growth of the older workforce*. Dec. 2023. URL: https://www.pewresearch.org/social-trends/2023/12/14/the-growth-of-the-older-workforce/#:~:text=Some%2019%25%20of%20adults%20ages,18%25%20of%20older%20Americans%20worked..

[48] Jr. John J. DiIulio et al. *Public service and the Federal Government*. June 2023. URL: https://www.brookings.edu/articles/public-service-and-the-federal-government/#:~:text=and%20small%20businesses.-,Across%20the%20U.S.%2C%20nearly%2024%20million%20people%E2%80%94a%20little%20over,in%20state%20and%20local%20governments..

[49] URL: https://www.bls.gov/careeroutlook/2017/article/older-workers.htm.

[50] Samuel Stebbins, Grant Suneson, and Douglas A. McIntyre. *These are the jobs with the oldest workforces in the United States, from farmers to shuttle drivers*. Oct. 2021. URL: https://www.usatoday.com/story/news/nation/2021/10/26/these-jobs-have-oldest-workforce-country/6166671001/.

[51] URL: https://www.lung.org/lung-health-diseases/lung-disease-lookup/asthma/learn-about-asthma/types/severe-asthma#:~:text=Diagnosing%20Severe%20Asthma&text=Of%20the%20more%20than%2025,or%20are%20just%20uncontrolled%20asthma..

[52] Eileen Wang et al. "Characterization of severe asthma worldwide: data from the International Severe Asthma Registry". In: *Chest* 157.4 (2020), pp. 790–804.

[53] URL: https://www.cancer.org/cancer/managing-cancer/side-effects/infections/preventing-infections-in-people-with-cancer.html.

[54] Eric S Donkor. "Stroke in the 21st century: a snapshot of the burden, epidemiology, and quality of life". In: *Stroke research and treatment* 2018.1 (2018), p. 3238165.

[55] Mohammed Yousufuddin and Nathan Young. "Aging and ischemic stroke". In: *Aging (Albany NY)* 11.9 (2019), p. 2542.

[56] URL: https://esrdnetworks.org/resources-news/national-esrd-census-data/#:~:text=National%20ESRD%20Data%20as%20of,%5Badd%20access%20date%20here%5D..

[57] Centers for Disease Control, Prevention, et al. "Chronic kidney disease in the United States, 2023". In: *Atlanta, GA: US Department of Health and Human Services, Centers for Disease Control and Prevention* (2023).

[58] URL: https://www.lung.org/about-us/our-impact#:~:text=More%20than%2035%20million%20people,living%20with%20a%20lung%20disease..

[59] Oct. 2024. URL: https://aafa.org/asthma/asthma-facts/#:~:text=Asthma%20can%20be%20deadly%20if,of%20Asthma%20Attacks%20in%20Children?.

[60] Hope Gillette. *Working with COPD: What are the options?* Jan. 2023. URL: https://www.healthline.com/health/can-you-work-with-copd.

[61] Nov. 2023. URL: https://www.cdc.gov/nchs/fastats/liver-disease.htm#:~:text=Number%20of%20adults%20age%2018,with%20diagnosed%20liver%20disease:%201.8%25.

[62] Daniela P Ladner et al. "Increasing prevalence of cirrhosis among insured adults in the United States, 2012–2018". In: *PloS one* 19.2 (2024), e0298887.

[63] URL: https://www.cff.org/intro-cf/about-cystic-fibrosis#:~:text=the%20United%20States:-,There%20are%20close%20to%2040%2C000%20children%20and%20adults%20living%20with,is%20age%2018%20or%20older..

[64] Anjali D Deshpande, Marcie Harris-Hayes, and Mario Schootman. "Epidemiology of diabetes and diabetes-related complications". In: *Physical therapy* 88.11 (2008), pp. 1254–1264.

[65] Taylor M Shockey, Rebecca J Tsai, and Pyone Cho. "Prevalence of diagnosed diabetes among employed us adults by demographic characteristics and occupation, 36 states, 2014 to 2018". In: *Journal of occupational and environmental medicine* 63.4 (2021), pp. 302–310.

[66] Anna Milanesi and Jane E Weinreb. "Diabetes in the elderly". In: (2015).

[67] Biykem Bozkurt et al. "Heart failure epidemiology and outcomes statistics: a report of the Heart Failure Society of America". In: (2023).

[68] Michael W Rich. "Heart failure in the 21st century: a cardiogeriatric syndrome". In: *The Journals of Gerontology Series A: Biological Sciences and Medical Sciences* 56.2 (2001), pp. M88–M96.

[69] URL: https://www.hiv.gov/hiv-basics/overview/data-and-trends/statistics#:~:text=At%20year%2Dend%202022%2C%20an,to%20the%20latest%20CDC%20data:.

[70] URL: https://www.hiv.gov/hiv-basics/living-well-with-hiv/taking-care-of-yourself/aging-with-hiv#:.

[71] Samuel D Emmerich et al. "Obesity and Severe Obesity Prevalence in Adults: United States, August 2021–August 2023". In: (2024).

[72] Yizhe Lim and Joshua Boster. "Obesity and comorbid conditions". In: (2021).

[73] URL: https://www.niaid.nih.gov/diseases-conditions/primary-immune-deficiency-diseases-pidds.

[74] URL: https://www.cdc.gov/primary-immunodeficiency/about/index.html#:~:text=Washing%20your%20hands%20the%20right,prior%20to%20treatment%20for%20SCID..

[75] Saramoriarty. *U.S. reaches historic milestone of 1 million transplants*. Oct. 2023. URL: https://unos.org/news/u-s-reaches-1-million-transplants/#:~:text=More%20than%20400%2C000%20people%20are%20alive%20today%20with%20a%20functioning%20transplant..

[76] URL: https://www.kidney.org.uk/are-work-and-a-normal-life-possible-after-a-transplant#:~:text=It%20is%20usual%20to%20take,directly%20on%20to%20the%20kidney..

[77] Paula M Williams. "Tuberculosis—United States, 2023". In: *MMWR. Morbidity and Mortality Weekly Report* 73 (2024).

[78] Aug. 2024. URL: https://www.dhs.wisconsin.gov/tb/precautions.htm#:~:text=Patients%20with%20confirmed%20infectious%20TB,is%20deemed%20to%20be%20noninfectious..

[79] Oct. 2024. URL: https://www.kff.org/other/state-indicator/distribution-by-age/?currentTimeframe=0&sortModel=%7B%22colId%22%3A%22Location%22%2C%22sort%22%3A%22asc%22%7D.
