# Supplementary material for: Protect or prevent? A practicable framework for the dilemmas of COVID-19 vaccine prioritization
Source: PLoS One. 2025 Jan 22;20(1):e0316294. doi: 10.1371/journal.pone.0316294 (PMC11753641; doi:10.1371/journal.pone.0316294)
Supplement: Attachment — Submitted filename: Response to Reviewers.pdf [file pone.0316294.s007.pdf]

Dear Editors,

I am writing on behalf of myself, Raghu Arghal, and my co-authors for our submission to PLOS One titled "Protect or prevent? A practicable framework for the dilemmas of COVID-19 vaccine prioritization". We greatly appreciate the time taken by both the editorial staff and the reviewer to provide useful feedback on our work. Your comments broached several valuable questions which have led to several changes that, in our view, considerably strengthen our work.

Briefly, we have substantially augmented our paper as follows:

1. Added Section 4.9 to address the reviewer's comment on overlap between high contact and high risk groups and SI 6 for data supporting Section 4.9.
2. Added a sensitivity analysis section (Section 4.5) and Figures 10 and 11 in it.
3. Relocated our robustness study from the SI to Section 4.6 with significant additions, including Figures 13 and 14.

In addition, we have included more details on (1) the evidence for group-specific contact rates and risk factors (see footnotes 2 and 3); and (2) our numerical methods (see red sentences in Section 3, page 11). For both we provide additional detail and relevant references.

We believe these changes address the suggestions given. Once again, we appreciate your consideration and the time taken to provide thoughtful feedback.

Below we further detail our responses to the reviewer's feedback pointy-by-point. For ease of reference, the reviewer's suggestions are reproduced in blue.

Thank you, and we look forward to hearing from you.

Reviewer 1:
Suggestions for minor revision:

1. **The overlap of the high contact and high risk groups**
"Authors should expand the Discussion on Group Overlaps: Provide a more detailed discussion on the potential overlap between high contact and high risk groups, including any assumptions or data supporting the claim that this overlap is minimal."
We have now significantly expanded the discussion on overlap between high contact and high risk groups. We have discussed several aspects of this overlap in a separately created subsection for the same (Section 4.9). The fact that this overlap is minimal throughout the world is objectively borne out from how closely our model, as shown in Section 4.1 based on the three group decomposition, predicts the temporal variation of infection and death counts which was observed in reality across the world. This is noted in Section 4.9. In addition, we have estimated that this overlap constitutes at most 1.9% of the US population from US census, employment data, CDC publications and health literature (SI 6). Note that we could not obtain an exact size of this overlap anywhere in the world including in US. We have mentioned the challenges in obtaining this exact size for US in SI 6. But, we could obtain an upper bound or an overestimate, we believe that the actual size of this overlap is significantly lower than this upper bound, as we have argued in SI 6.
Since however this overlap is positive, and can not be reliably estimated all over the world, we have also augmented our model with a fourth group which comprises of the overlap, that is, it is *both* high contact and high risk. We numerically study this augmented model over ∼5.5M instances, while varying contact rates, group sizes of all four groups, COVID variants, R_0, NPI efficacy, and initial infection rate. Across all these instances, we find that the fourth group is *always* given first priority for vaccination by the optimal policy. Intuitively this is because individuals in this group spreads the disease at the highest rate while they are in the states in which individuals can spread the disease (ie, infectious but not hospitalized or dead) and they have the highest risk of developing serious forms of the disease leading to hospitalization and death. Since usually the fourth group is small in size the vaccination process for such individuals is concluded in a short time if they are accorded the first priority as the optimal policy does. Subsequently, the remaining individuals ought to be vaccinated, whose relative priority can be determined by the optimal policy by solving the optimal control problem formulated for the three groups as in Section 3 and which has been reported in

Sections 4.2 to 4.7. Thus for a wide range of parameters seen for COVID the optimal vaccination policy of four groups can be obtained through a simple and intuitive augmentation of that obtained for three groups. These findings substantiate the intuition that we had provided in our initial submission:

> "In principle, the vaccination priority for the small fraction of individuals who are both high contact and high risk ought to be the highest. Thus, they can be excluded from the decision problem we consider, i.e. we consider the problem of allocation of vaccines to individuals after those who are both high contact and high risk are vaccinated."

2. **Sensitivity analysis**

   (a) "To better understand the robustness of the model's outcomes, i recommened, if posssible, the authors include a sensitivity analysis to explore how changes in key parameters (e.g., contact rates, vaccine efficacy, or group sizes) might affect results. This would help determine the model's reliability under different scenarios."
   We have now expanded our robustness study, along the directions suggested by the reviewer, and have moved it from the SI to Section 4.6 of the manuscript. As suggested by the reviewer, we have now explained the robustness findings through a sensitivity analysis to explore how changes in key parameters (e.g., contact rates, vaccine efficacy, or group sizes) might affect the optimum vaccination policy. Refer to the paragraphs in red in Section 4.6 and Figures 13 and 14.

   (b) "Authors should incorporate Sensitivity Analysis: Add a section on sensitivity analysis to explore how different parameter values might affect the model outcomes."
   We have now added a Section 4.5 on Sensitivity Analysis, including Figures 10 and 11, to explore how different parameter values, specifically the parameters the reviewer suggested, e.g., contact rates, vaccine efficacy, or group sizes, affect model outcomes such as composition of the optimum vaccination policy and the optimum value of the objective function, namely the optimum death count. The sensitivity analysis in this Section is somewhat different from that used to explain robustness in Section 4.6; we point out the difference in Section 4.6. We also briefly summarize these additions in our Discussion (red sentences and phrases in Section 5, page 33).

3. **Group-specific contact rates and risk factors evidence**
   "Authors should include Empirical Data or References: Strengthen the rationale for group-specific contact rates and risk factors with empirical data or references to existing studies."
   We have now strengthened our rationale for group-specific contact rates and risk factors using example empirical data obtained from existing studies for individuals belonging to different professions and age groups. The data we have now produced show that mortality risks can substantially increase due to age or underlying diseases (see footnote 2). Thus, mortality risks would be different for different groups if groups are formed based on age or underlying disease or lack thereof (this is how we form our three groups, as described in Section 3, paragraphs 1, 2 of the submitted paper). The data we have now produced also show that 1) contact rates vary widely depending on professions and age, and 2) contact rates across age groups can significantly differ from those within age groups. Please refer to footnote 3. Note that we calculated contact rates within and across the groups we formed, as per the methodology described in the second and third paragraphs of Section 4.1 and SI 4.1 of the submitted manuscript. We obtained risk factors (specifically probabilities of symptomatic infection, hospitalization, death) for different groups from quoted sources, as described in SI 1. With these parameters and group structure, our model closely predicted the temporal variation of infection and death counts which was observed in reality in all 50 states of US and 139 countries overall (Section 4.1). This also justifies our use of group specific contact rates and risk factors.

4. **Numerical methods clarity**
   "Authors should explanation of Numerical Methods: The methodology mentions using numerical toolboxes Yop and CasADi for solving the optimal control problem. However, it lacks a detailed description of how these tools are used, including any specific algorithms or settings. Providing this detail would make the methodology more reproducible."
   We have included more information on the exact method we employ to solve the optimal control problem along with an additional reference which details the method (see red sentences in Section 3, page 11).

Sincerely,

Raghu Arghal
rarghal@upenn.edu
